# Financial burden of postoperative complications following colonic resection: A systematic review

**DOI:** 10.1097/MD.0000000000026546

**Published:** 2021-07-09

**Authors:** Maleck Louis, Samuel A. Johnston, Leonid Churilov, Ronald Ma, Christopher Christophi, Laurence Weinberg

**Affiliations:** a Department of Anesthesia, Austin Health, Victoria, Australia; b Department of Anesthesia, Austin Health, Victoria, Australia; c Department of Medicine (Austin Health) & Melbourne Brain Centre at Royal Melbourne Hospital, Melbourne Medical School, Faculty of Medicine, Dentistry and Health Sciences, Victoria, Australia; d Department of Finance, Austin Health, Victoria, Australia; e Department of Surgery, The University of Melbourne, Austin Health, Victoria, Australia; f Department of Anesthesia, Austin Health, Heidelberg, Australia; g Department of Surgery, The University of Melbourne, Austin Health, Victoria, Australia.

**Keywords:** colon surgery, complication, cost, cost analysis

## Abstract

**Background:**

Colonic resection is a common surgical procedure that is associated with a high rate of postoperative complications. Postoperative complications are expected to be major contributors to hospital costs. Therefore, this systematic review aims to outline the health costs of postoperative complications following colon resection surgery.

**Methods:**

MEDLINE, Excerpta Medica database, Cochrane, and Economics literature medical databases were searched from 2010 to 2019 to identify English studies containing an economic evaluation of postoperative complications following colonic resection in adult patients. All surgical techniques and indications for colon resection were included. Eligible study designs included randomized trials, comparative observational studies, and conference abstracts.

**Results:**

Thirty-four articles met the eligibility criteria. We found a high overall complication incidence with associated increased costs ranging from $2290 to $43,146. Surgical site infections and anastomotic leak were shown to be associated with greater resource utilization relative to other postoperative complications. Postoperative complications were associated with greater incidence of hospital readmission, which in turn is highlighted as a significant financial burden. Weak evidence demonstrates increased complication incidence and costlier complications with open colon surgery as compared to laparoscopic surgery. Notably, we identified a vast degree of heterogeneity in study design, complication reporting and costing methodology preventing quantitative analysis of cost results.

**Conclusions:**

Postoperative complications in colonic resection appear to be associated with a significant financial burden. Therefore, large, prospective, cost-benefit clinical trials investigating preventative strategies, with detailed and consistent methodology and reporting standards, are required to improve patient outcomes and the cost-effectiveness of our health care systems.

## 1 Introduction

### 1.1 Rationale

Cost-effective health care, particularly in the hospital setting, is crucial for the sustainability of our health care systems. On the international level, health care expenditure has increased at a faster annual rate than economy growth between the years 2000 and 2016.^[1]^ Rising health care costs combined with the continual necessity for high quality care, has resulted in growing demand by policy makers for high quality health economic assessments.

Vonlanthen et al^[2]^ report that postoperative complications are the strongest indicators of in-hospital costs. These findings were reinforced by a systematic review demonstrating increased hospital costs from surgical complications after major surgery.^[3]^ Limitations of this review were that it failed to report colorectal complications. Given that colon resection surgery is a common procedure with a high rate of postoperative adverse events relative to other major surgeries,^[4,5]^ it is expected to be a major contributor to hospital costs. The development of cost-effective management strategies targeting colon resection surgery is dependent on accurate financial data and a deep understanding of the relationship between postoperative complications and the drivers of increased hospital costs.

### 1.2 Objectives

The primary aim of this systematic literature review is to outline the health costs of postoperative complications in adult patients who undergo colon resection surgery. We highlight the importance of evaluating the components of healthcare cost profiles relevant to patients undergoing colon resections and consider the quality of the studies with reference to how they measure and report costing data.

## 2 Materials and methods

### 2.1 Protocol and registration

We conducted a systematic review of the literature in accordance with Cochrane guidelines^[6]^ and reported under the guidance of the preferred reporting items for systematic reviews and meta-analyses statement.^[7]^ The protocol of this review was registered in PROSPERO, an international prospective register of systematic reviews, and is available from: http://www.crd.york.ac.uk/PROSPERO/display_record.php?ID=CRD42019128618. The Austin Health Research Ethics Committee waivered the requirement for ethics approval as collection of data did not involve patient contact or patient data.

### 2.2 Eligibility criteria

We included studies containing a full or partial economic evaluation of postoperative complications in adult patients (≥18 years of age) undergoing any form of colonic resection. Colon resection was defined as complete excision of any part of the large bowel (excluding rectum). Studies that did not report the cost of colon resection specifically were excluded.

Eligible study designs included randomized controlled trials (RCTs), non-RCTs, comparative observational studies and conference abstracts. Letters, opinion papers and editorials were excluded. Only studies in the English language were considered and no restriction by country or currency was applied.

Enhanced recovery after surgery^[8]^ was established globally and adopted by many hospitals as a uniform approach to minimizing variability of perioperative care. Taking this recent advancement into account, we included studies published from January 2010 until February 2019 in order to retrieve up to date cost data.

### 2.3 Primary outcome

Total hospital costs associated with complications following colonic resection surgery

### 2.4 Secondary outcomes

(1) The costs of individual complications after colonic surgery (e.g., anastomotic leak, surgical site infection [SSI] etc)
(2) The association of severity of complications and hospital costs.
(3) The impact of complication on length of hospital stay, mortality and 30-day readmission rates.
(4) Costs associated with postoperative complications by surgical technique.
(5) Costs associated with postoperative complications by indication for surgery.
(6) Costs associated with postoperative complications by surgical urgency (emergent or elective).

### 2.5 Information sources and search

A detailed search strategy was constructed based on the topic title. The search was conducted by one author (ML) and applied to Economics literature (EBSCOhost; January 2010–present), MEDLINE (Ovid; 2010 - present), Excerpta Medica database (Ovid; 2010 - present) and The Cochrane Library (Wiley Online Library; 2010 - present). The last search was run on 19/02/2019. The search strategy is described in Table S1, Supplemental Digital Content. Medical subject Headings terms and free-text terms on costs, health economics, colonic resections and complications were used. An additional manual search of the bibliographies of all identified studies and review articles was also performed.

### 2.6 Study selection

The titles and abstracts of all retrieved studies were screened by 2 authors (ML and SJ) in an independent and blinded manner. The full texts of eligible studies were retrieved and independently evaluated for eligibility by 3 authors (ML, SJ, and LW). Disagreements were resolved by a fourth author (RM) and by consensus.

### 2.7 Data collection process and data items

Data from included studies was extracted in an independent manner by 2 authors (ML and SJ) into a predetermined data extraction table. Extracted data included study characteristics, colon resection procedure and technique, population demographics, complication incidence, cost of complications, length of stay (LOS), mortality, 30-day readmission, indication for colonic resection, and urgency status. A copy of the data extraction table is presented in Tables S2 and S3, Supplemental Digital Content.

### 2.8 Risk of bias in individual studies

Risk of bias of the included studies were assessed by 2 authors (ML and SJ) using the Cochrane Collaboration's risk of bias tool to assess randomized controlled trials (RCTs) and the Scottish Intercollegiate Guidelines Network (SIGN) Checklist for Cohort Studies to assess cohort studies. Discrepancies were resolved by consensus following review by a third author (LW).

### 2.9 Summary measures and synthesis of results

Findings are reported in the form of a narrative synthesis. This is structured around the type of complications and their hospital costs. Cost of complications was derived from either the stated value within the study or by calculation of the cost difference between the group with and the group without complications. Costs were converted to United States dollar (USD) ($) based on the annual average conversion rate^[9]^ of the specified base currency year or the year of publication if a currency year was not reported. Costs were then inflated to February 2019 from January of the specified or assumed cost year using the Bureau of Labor Statistics Consumer Price Index inflation calculator.^[10]^

We referenced complication costs to the complication type, complication severity, surgical technique, indication for surgery, surgical urgency, readmissions, mortality, and LOS. A critical review of the data showed significant discrepancies in economic environment and hospital characteristics of the included studies resulting in significant heterogeneity of the studies, therefore a meta-analysis could not be performed.

### 2.10 Risk of bias across studies

Homogenous effect sizes across studies were unavailable. Therefore, formal assessment of publication bias using a funnel plot was not presented. Each of the reported study's outcomes and results were compared to assess for selective reporting bias.

### 2.11 Additional analyses

Subgroup analyses were undertaken for resource utilization measure, readmissions, surgical technique, number and severity of complications and type of complication.

## 3 Results

### 3.1 Study selection

**Figure 1:**
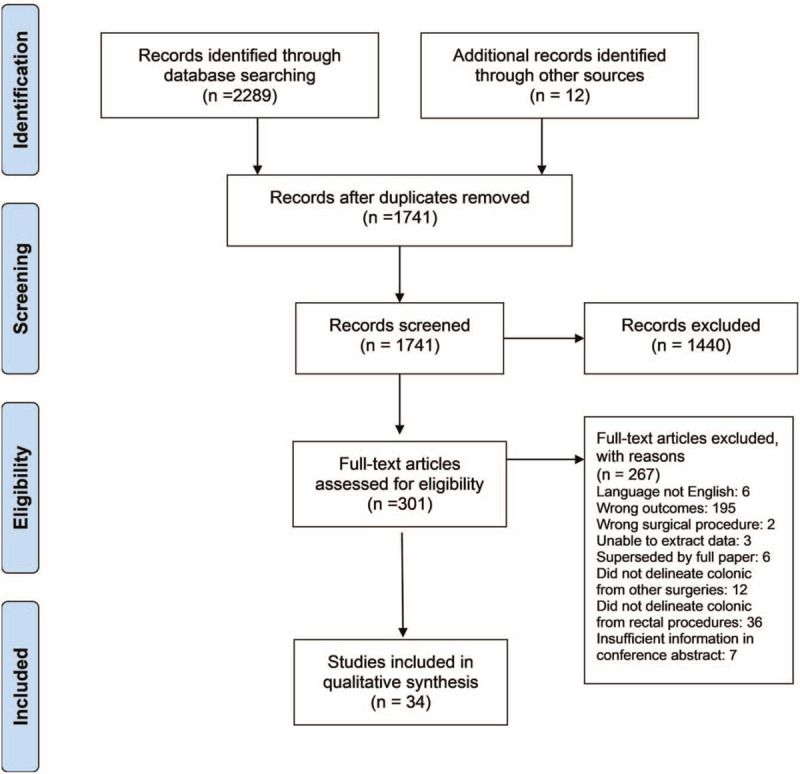
PRISMA flow diagram summarizing the study selection process of the systematic review. PRISMA = preferred reporting items for systematic reviews and meta-analysis.

The search strategy resulted in a total of 2289 articles being sourced. Twelve additional studies were identified by manual searching of bibliographies. Thirty-four articles^[11–44]^ met the eligibility criteria. The preferred reporting items for systematic reviews and meta-analyses flow diagram^[7]^ representing the selection of studies is presented in Figure 1.

### 3.2 Study characteristics

All included studies were reported in English and had publication dates between 2010 and 2018. Thirty-one studies were retrospective cohort studies,^[11–13,15–28,30–33,35–44]^ with the remaining studies consisting of a cross-sectional cohort study,^[34]^ an RCT^[29]^ and a randomized clinical trial^[14]^. Twenty-five studies were full reports^[11,12,14,16–20,22,24,25,27–29,31,32,34,35,37,38,40–44]^ and 9 were conference abstracts.^[13,15,21,23,26,30,33,36,39]^ Study characteristics and outcome measures are presented in Tables S2 and S3, Supplemental Digital Content.

### 3.3 Population

Number of participants ranged from 46 patients^[29]^ to 217,939 patients.^[41]^ Reported mean and median patient age ranged from 49.9 years^[24]^ to 78.5 years.^[43]^ Most studies incorporated broad inclusion criteria, including all colon resections performed within the specified time frame. Details on patient demographics are presented in Table S2, Supplemental Digital Content.

### 3.4 Primary outcome

Twenty-one studies^[11,13,15–22,25–28,33,36,38–41,44]^ evaluated cost of complications in colonic resection as one of their key outcomes. Thirteen articles^[12,14,23,24,29–32,34,35,37,42,43]^ did not study the cost of complications as a key outcome but contained sufficient complication and financial data to meet inclusion criteria.

### 3.5 Defining currency

Twenty-nine studies^[11–13,15–25,27,28,30–34,36–38,40–44]^ reported costs using USD ($), three used Euro (€)^[14,26,29]^ and one used New Zealand Dollars ($)^[35]^. One study^[39]^, a conference abstract, did not report the currency used. This was assumed to be USD due to the study being conducted in New York, USA. Only 8 studies^[16–18,27,31,34,40,41]^ reported the base currency year.

### 3.6 Defining hospital resource utilization

Nineteen studies^[11,13–15,17,19–22,25,28–30,32,33,35,36,40,41]^ reported hospital costs, with twelve studies^[12,16,23,24,27,31,34,38,39,42–44]^ reporting total hospital reimbursements, two studies^[18,37]^ reporting hospital charges and one study^[26]^ reporting negative Diagnosis Related Groups based cost coverage. Where multiple hospital resource utilization measures were reported, hospital costs were extracted. The definition of hospital resource for each study is presented in Table S2, Supplemental Digital Content.

### 3.7 Risk of bias within studies

Thirty-one out of the thirty-four included studies were retrospective cohort studies with their retrospective nature preventing them from being considered “high quality” evidence based on the SIGN cohort study checklist. All twenty-two retrospective cohort full paper publications^[11,12,16–20,22,24,25,27,28,31,32,35,37,38,40–44]^ were of “acceptable quality” with clear focused research questions. The risk of bias in the nine conference abstracts^[13,15,21,23,26,30,33,36,39]^ was not formally assessed due to the incomplete nature of their reporting. The cross-sectional cohort study^[34]^ was deemed of “acceptable quality” as it is classified as a database study by the SIGN cohort study checklist. The overall risk of bias for the randomized controlled^[14]^ and the randomized clinical trial^[29]^ was judged to be low with satisfactory randomization and no evidence of selection bias.

### 3.8 Risk of bias across studies

Noninclusion of grey literature in this review may have resulted in publication bias. In addition, inclusion of studies only in the English language may have resulted in a language bias. The principally objective nature of the financial and clinical outcomes assessed in this review makes outcome measurement an unlikely bias. Conference abstracts are at a high risk of selective reporting bias and this was considered in our data synthesis.

*Synthesis of results*: all costs are presented as: study reported costs (adjusted USD cost).

### 3.9 Incidence and cost of complications

**Figure 2:**
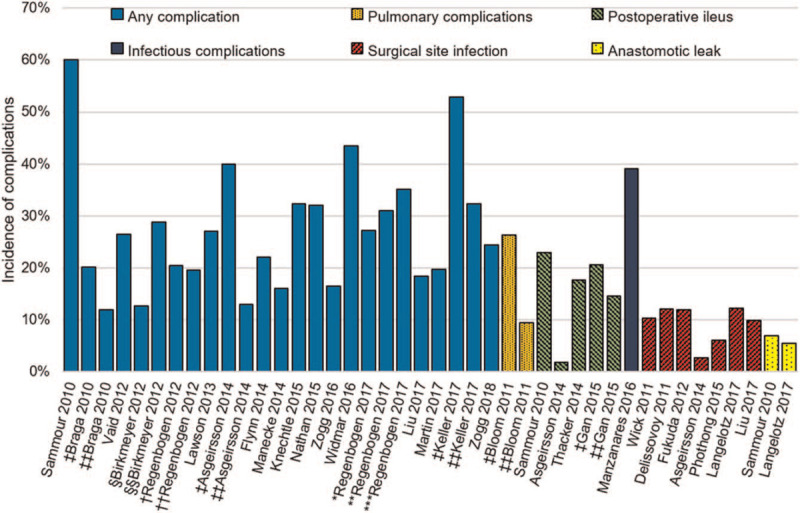
The incidence of postoperative complications (%) following colonic resection surgery, stratified by complication type when available, compared across studies ^‡^Open, ^‡‡^Laparoscopic; Surgical volume: ^†^Low, ^††^High; Hospital quality: ^§^Lowest, ^§§^Highest; Hospital length of stay: ^∗^≤3, ^∗∗^4–6, ^∗∗∗^≥7 days.

**Figure 3:**
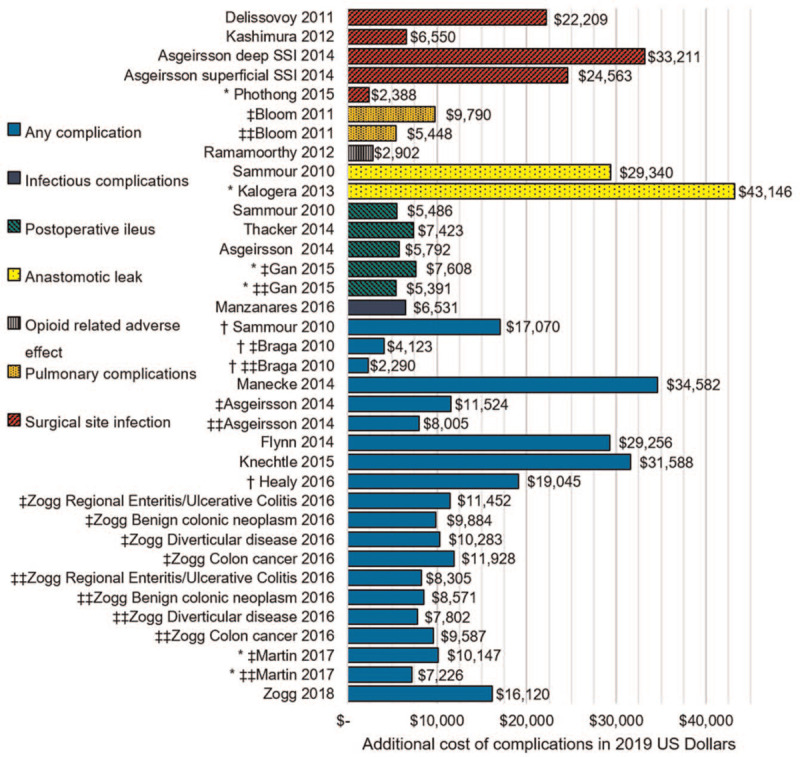
The increase in hospital costs due to postoperative complications following colonic resection surgery, stratified by complication type when available, compared across studies. Costs are reported in 2019 USD ($) and are presented as mean unless otherwise stated ^∗^ Median, ^†^Unclear; ^‡^Open, ^‡‡^Laparoscopic.

Postoperative complication incidence varied greatly between the studies ranging from 6.0%^[32]^ to 66.0%^[35]^ (Fig. 2). This variance can be attributed to the different definitions of complications adopted by the studies. Studies reporting hospital costs were analyzed separately to studies reporting hospital charges or hospital reimbursements. Postoperative complications resulted in a substantial increase in hospital costs across all studies (Fig. 3). The additional costs of complications varied from €1478.63 [$2290]^[14]^ to $39,306 [$43,146],^[21]^ this is in part due to the heterogenous definitions of hospital costs adopted by the different studies as well as the different complication types reported. Asgeirsson et al^[11]^ and Knechtle et al^[25]^ further demonstrate a positive correlation between the count of complications and the additional cost incurred by the hospital. All studies reporting hospital charges and hospital reimbursements demonstrated a positive increase in hospital resource utilization with postoperative complications (Table S2, Supplemental Digital Content).

### 3.10 Complication severity

Asgeirsson et al^[11]^ demonstrated an increase in the additional cost of SSI from $22,730 [$24,563] for superficial SSIs to $30,733 [$33,211] for deep SSIs. Fukuda et al,^[18]^ demonstrated similar increases in hospital charges with increasing SSI severity. Widmar et al^[39]^ analyzed the impact of complication severity on Medicare reimbursements by utilizing the Clavien-Dindo classification. Thirty-day hospital reimbursements increased from $3520 [$3756] for no complication to $5570 [$5943] and $7,610 [$8119] for grade I and grade II complications, respectively.^[39]^ There was an exponential rise in reimbursements to $17,124 [$18,270] for grade III+ complications.^[39]^ Despite this, grade I and grade II complications remain a significant health care burden due to their high prevalence rates (29% and 49%, respectively).^[39]^

### 3.11 Cost by complication type

SSI, postoperative ileus, and anastomotic leak were the most commonly reported postoperative complications. Results and outcome metrics of studies reporting the financial burden of these complications are presented in Table 1. SSI and anastomotic leak were associated with the greatest financial burden amongst postoperative complications in colon resection surgery. The additional hospital cost of SSI varied greatly across studies (Table 1). This significant variation in costs for SSI can be attributed to the geographic differences in health care systems. Asgeirsson et al^[11]^ was the only study directly comparing SSI with postoperative ileus demonstrating significantly higher additional costs in the presence of SSI than with postoperative ileus (Table 1).

**Table 1:** Summary of studies evaluating the financial burden of surgical site infection, postoperative ileus and anastomotic leak following colonic resection surgery.

Author, year, reference number	Study design	Country, currency and price year	Patients (n) and dates of patient sample	Surgery	Outcome definition	Incidence and cost of complications
Surgical site infection						
Delissovoy et al^[15]^	Multicenter retrospective cohort study utilizing the Premier Perspective Comparative Database *(Conference abstract)*	USA; USD ($) assumed 2011	Number of patients undergoing colon procedures not reported; 2007–2010	Colon resection; Surgical technique not specified	Costs: Hospital costs. Definition not reported. Postoperative complications SSI was identified by a combination of postoperative infection diagnosis codes, or postoperative prescription of selected antimicrobial drugs with treatment duration ≥5 days.	Incidence (95%CI) of SSI = 12.0%, (11.78–12.2%) Mean (95%CI) additional cost of SSI = $19,349 (19,315–19,383) **[$22,209]**
Wick et al^[38]^	Multicenter retrospective cohort study utilizing Blue Cross and Blue Shield insurance plan claims database	USA, USD ($) assumed 2011	7020 patients undergoing partial or total colectomy; January 2002–December 2008	laparoscopic colon resection = 1273 (18.1%) Open colon resection = 5747 (81.9%)	Costs: Hospital reimbursements defined as payments to hospitals from day of operation to 90 days postoperatively. Postoperative complications SSI was identified using ICD-9-CM codes.	Incidence of SSI = 726/7020 (10.3%) Mean (95%CI) reimbursement for SSI: With SSI = $31,933 (29,607–34,258) **[$36,653]** Without SSI = $14,608 (14,018–15,197) **[$16,767]** ; *P*-value < .001
Fukuda et al^[18]^	Multicenter retrospective cohort study utilizing the Diagnosis Procedure Combination/ Per-Diem Payment System and Japan Nosocomial Infections Surveillance databases	Japan; USD ($) 2010 Converted and adjusted from Yens (US$1 = ¥122.1)	1817 patients out of which 1308 patients were undergoing colon resection; September 2007 to December 2010 *Only 1108 colon resection patients had data for postoperative resource consumption*	Laparoscopic colon resection = 381 (29.1%) Open colon resection = 927 (70.9%)	Costs: Hospital charges defined as expenditure incurred between the first day postoperatively and the day of discharge (surgery charges on the day of operation were not included) calculated by multiplying the volume of resources consumed per patient by the official unit price. Postoperative complications: SSI was identified using the standard Centers for Disease Control and Prevention National Nosocomial Infections Surveillance System criteria.	Incidence of SSI = 156/1308 (11.9%) • Superficial SSI = 73/1108 (6.6%) • Deep SSI = 15/1108 (1.4%) • Space/organ SSI = 9/1108 (0.8%) Mean (95% CI) charge of SSI • With SSI = $4189 (4114–4266) **[$4,887]** • Without SSI = $2,973 (2919–3033) **[$3,468]** ; *P*-value < .001 Mean (95%CI) additional charge of SSI = $1216 (1196–1240) **[$1419]**
Kashimura et al^[22]^	Multicenter retrospective-matched cohort study	Japan; USD ($) assumed 2012	334 patients out of which 204 patients underwent colon resection; April 2006–March 2008	Open colon resection = 154 (75.5%) laparoscopic colon resection = 50 (24.5%)	Costs: Hospital costs, defined as cost of index admission postsurgery, plus any costs incurred during readmission secondary to SSI. Costs were calculated using the fee-for-service calculation method. Postoperative complications SSI was identified using Centers for Disease Control and prevention criteria.	Case-matched population with 102 patients with SSI Mean (SD) cost of SSI • With SSI = $10,152 (13,474) **[$11,322]** • Without SSI = $4279 (2945) **[$4,772]** Mean (95%CI) additional cost of SSI = $5873 (3166–8579) **[$6550]**
Asgeirsson et al^[11]^	Single-center retrospective cohort study	USA; USD ($) assumed 2014	1422 patients undergoing segmental colectomy; July 2008–June 2012	Laparoscopic segmental colectomy = 654 (46.0%) Open Segmental colectomy = 768 (54.0%)	Costs: Hospital costs derived from the institutional cost accounting system for 30 days postsurgery Postoperative complications: SSI was defined as superficial, deep, and organ space (in the absence of anastomotic leak).	Incidence of SSI = 39/1422 (2.7%) • Superficial Site Infection = 12/1422 (0.8%) • Deep Surgical Site infection/Organ space = 27/1422 (1.9%) Mean (SD) additional cost of SSI • Superficial SSI = $31,899 (14,705) **[$34,471]** • Deep SSI/organ space = $39,902 (27,911) **[$43,119]**
Phothong et al^[32]^	Single-center retrospective cohort study	Thailand; USD ($) assumed 2015	100 case-matched patients undergoing sigmoidectomy; January 2008–September 2013	Hand-assisted laparoscopic sigmoidectomy = 50 (50%) Open sigmoidectomy = 50 (50%)	Costs Hospital costs including room charges, operating room costs, anesthesia costs, instrument costs and other hospital costs. Postoperative complications SSI definition not reported.	Incidence of SSI = 6/100 (6.0%) *Cost data available for SSI in open surgery group only* Median (range) cost of SSI • With SSI = $4348 ($2185–$11,509) **[$4703]** • Without SSI = $2140 ($1379–$6277) **[$2315]** ; *P*-value = 0.004
Langelotz et al^[26]^	Single-center retrospective cohort study utilizing the Institute for the Hospital Remuneration System (Germany) database *(Conference abstract)*	Germany, Euro (€) assumed 2017	460 patients undergoing colonic resections; 2010–2015	Colonic resection; surgical technique not specified	Costs: Cost coverage effect of complications. Cost definition not reported. Postoperative complications SSI were identified using DRG codes.	Incidence of SSI = 56/460 (12.2%) Median negative cost coverage effect of complications Without SSI = –668€ **[–$786]** With SSI only = –6823€ **[–$8026]**
Liu et al^[27]^	Multi-center retrospective cohort study utilizing the ACS-NSQIP and the Medicare Provider Analysis and Review databases.	USA; USD ($) 2012	19,089 patients undergoing elective colectomy; 2009–2012	Colectomy; Surgical technique not specified	Costs: Payments to hospitals by Centers for Medicare and Medicaid Services based on MS-DRG categories. Payments not directly related to care were excluded. Postoperative complications Complications were identified using ACS-NSQIP criteria.	Incidence of SSI = 9.8% Mean (95%CI) reimbursement for SSI With SSI = $16,257.21 (15,940.01–16,580.72) **[$18,130]** Uncomplicated = $13531.16 (13,390.63–13,673.15) **[$15,090]** Additional reimbursement for SSI = $2726.05 **[$3040]**
Postoperative Ileus						
Sammour et al^[35]^	Single-center retrospective cohort study ERAS group was collected prospectively Control group was collected retrospectively	New Zealand, NZD ($) assumed 2010	100 patients undergoing elective colonic surgery; • ERAS program: 50 consecutive patients (December 2005–March 2007) • Pre-ERAS program: 50 consecutive patients (September 2004–September 2005)	Open and laparoscopic colon resection techniques included and analyzed as one group	Costs: Hospital costs defined as cost of index hospital stay excluding cost of day stay and readmissions. Postoperative complications POI definition not reported.	Incidence of POI = 23/100 (23.0%) Additional cost of POI = $6517.37 **[$5,486]**
Asgeirsson et al^[11]^	Single-center retrospective cohort study	USA; USD ($) assumed 2014	1422 patients undergoing segmental colectomy; July 2008–June 2012	Laparoscopic colectomy = 654 (46.0%) Open colectomy = 768 (54.0%)	Costs: Hospital costs derived from the institutional cost accounting system for 30 days post-surgery Postoperative complications: Postoperative ileus definition not reported.	Incidence of POI = 26/1422 (1.8%) Mean (SD) additional cost of POI = $14,529 (12,953) **[$15,700]**
Thacker et al^[36]^	Multicenter retrospective cohort study utilizing the Premier research database (US) *(Conference abstract)*	United Kingdom; USD ($) assumed 2014	84,722 patients undergoing elective colon surgery; January 2008–June 2012	Colon surgery; Surgical technique not specified.	Costs: Hospital costs Definition not reported Postoperative complications POI identified using ICD-9-CM codes.	Incidence of POI = 14,972 (17.7%) Mean (SD) total hospital costs With POI = $20,734 (14,506) **[$22,406]** Without POI = $13,865 (8,315) **[$14,983]** ; *P*-value < .001
Gan et al^[19]^	Multicenter retrospective cohort study utilizing The Premier Research Database	USA; USD ($) assumed 2015	138,068 patients out of which 57,948 patients were undergoing colon resection; September 2008 – August 2010	Open colon resection = 40,250 (69.5%) Laparoscopic colon resection = 17,698 (30.5%)	Costs: Hospital costs derived from all billed items at the individual patient level. Postoperative complications POI identified using ICD-9-CM codes.	Incidence of POI = 10,880/57,948 (18.8%) Median cost for laparoscopic colon resection: With POI = $17,505 [**$18,933]** Without POI = $12,521 **[$13,543]** ; *P*-value < .001 Median cost for open colon resection: • With POI = $24,078 **[$26,043]** • Without POI = $17,044 **[$18,435]** ; *P*-value < .001
Anastomotic Leak						
Sammour et al^[35]^	Single-center retrospective cohort study ERAS group was collected prospectively Control group was collected retrospectively	New Zealand, NZD ($) assumed 2010	100 patients undergoing elective colonic surgery; • ERAS program: 50 consecutive patients (December 2005–March 2007) • Pre-ERAS program: 50 consecutive patients (September 2004–September 2005)	Open and laparoscopic colon resection techniques included and analyzed as *one group*	Costs: Hospital costs defined as cost of index hospital stay excluding cost of day stay and readmissions. Postoperative complications Anastomotic leak definition not reported.	Incidence of anastomotic leak = 7/100 (7.0%) Additional cost of anastomotic leak = $34,853.26 **[$29,340]**
Kalogera et al^[21]^	Single-center retrospective cohort study *(Conference abstract)*	USA, USD ($) assumed 2013	42 Anastomotic leak cases matched with 84 no-leak controls undergoing large bowel resection for primary ovarian cancer; 1994–2011	Large bowel resection; Surgical technique not specified	Costs: Hospital costs excluding outpatient cost data. Postoperative complications Anastomotic leak definition not reported.	Case-matched population with 42 patients with anastomotic leaks Median (IQR) cost of anastomotic leak at 30 days • With anastomotic leak = $72,760.4 (52,858.9–104,449.2) **[$79,868]** • Without anastomotic leaks = $33,453.7 (27,081.0–41,743.0) **[$36,722]** ; *P*-value <.001
Langelotz et al^[26]^	Single-center retrospective cohort study utilizing the Institute for the Hospital Remuneration System database *(Conference abstract)*	Germany, Euro (€) assumed 2017	460 patients undergoing colonic resections; 2010–2015	Colonic resection; surgical technique not specified	Costs: Cost coverage effect of complications. Cost definition not reported. Postoperative complications Anastomotic insufficiency was identified using DRG codes.	Incidence of anastomotic insufficiency = 25 (5.4%) Median negative cost coverage effect of complications: Without anastomotic insufficiency = –668€ **[**–**$786]** With anastomotic insufficiency only = –2659€ **[**–**$3128]**

Costs in bold, **[$$$]**, have been converted and inflated to February 2019 $USD from assumed cost year. 95% CI = 95% confidence interval, ACS-NSQIP = American College of Surgeons-National Surgical Quality Improvement Project, DRG = diagnosis related group, ERAS = enhanced recovery after surgery, ICD-9-CM = International Classification of Diseases, Ninth Revision, Clinical Modification, IQR = interquartile range; MS-DRG = Medicare Severity - Diagnosis Related Group, NZD = New Zealand Dollar, POI = postoperative ileus, SD = Standard Deviation, SSI = surgical site infection, USD = United States Dollar.

### 3.12 Cost of surgical technique

Nine studies^[11,13,14,18,19,22,30,37,40]^ reported the cost of complications in open and laparoscopic surgery, however only one study^[37]^compared the two groups as its primary outcome. Postoperative complications in open surgery were shown to be associated with higher hospital costs than postoperative complications in laparoscopic surgery across all included studies except Kashimura et al^[22]^ (Table S3, Supplemental Digital Content). In addition, the incidence of complications was consistently higher in open surgery as compared to laparoscopic surgery in all studies, but only reached statistical significance in six studies.^[11,18,24,37,38,40]^ The risk of selection bias due to the retrospective nature of the studies and existence of uncertainty around the statistical significance of data reported in some studies means that these findings are supported by weak evidence.

### 3.13 Cost of readmissions

**Figure 4:**
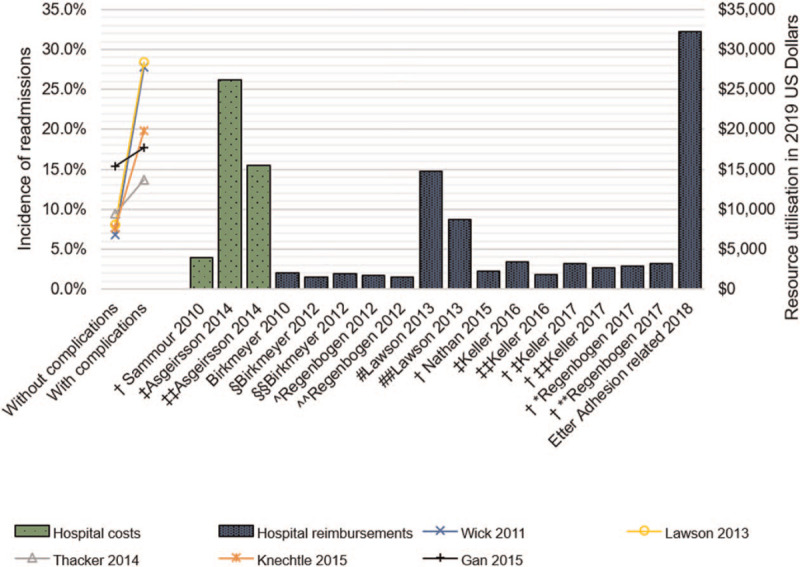
The incidence of readmissions in the presence and absence of postoperative complications following colonic resection surgery with readmission resource utilization compared across studies. Resource utilization is reported in 2019 USD ($) and is presented as mean unless otherwise stated ^†^Unclear; ^‡^Open, ^‡‡^Laparoscopic; Surgical volume: ^^^Low, ^^^^High; Hospital quality: ^§^Lowest, ^§§^Highest; Hospital length of stay: ^∗^≤3, ^∗∗^≥7 days, Complication: ^#^Yes, ^##^No.

Incidence of readmissions ranged from 6.6%^[23]^ to 28.4%^[44]^ with greater readmission rates for patients who experienced postoperative complications in the index admission as compared to patients who had an uncomplicated admission (Fig. 4). Reimbursement for readmissions varied greatly across the studies, from $1322 [$1474]^[12]^ to $29,802 [$32,234]^[16]^ (Fig. 4).

### 3.14 Length of stay

Postoperative complications resulted in an increased hospital LOS across all studies with additional LOS ranging from 1.5 days^[38]^ to 19 days.^[26]^ In addition, the greater the cumulative number of complications a patient experienced, the greater their hospital LOS.^[25]^ Increasing SSI severity was also associated with increased hospital LOS.^[18]^ No study assessed the direct cost impact of LOS.

### 3.15 Mortality

Three studies^[28,36,40]^ reported increased mortality rates associated with incidence of postoperative complications. No study reported the cost impact of mortality.

### 3.16 Indication for surgery

Impact of indication for surgery on incidence of complications was inconclusive across all studies.^[18,38,40]^ Zogg et al^[40]^ was the only study assessing the cost impact of indication for surgery demonstrating increased costs associated with patients with colon cancer experiencing postoperative complications.

### 3.17 Surgical urgency

Impact of surgical urgency on incidence and cost of complications is inconclusive across all studies.^[11,17]^ Fukuda et al^[18]^ reported no statistical association between surgical urgency and risk of SSI. However, Asgeirsson et al^[11]^ reported higher complication incidence and hospital costs with urgent/emergent admissions as compared to elective admissions.

### 3.18 Cost breakdown

Only two studies^[32,38]^ reported the financial burden associated with complications broken down into different hospital cost centers (Table S2, Supplemental Digital Content). Both studies explored the costs associated with SSI, demonstrating the greatest cost difference in inpatient costs^[38]^ and “room,” “operative,” and “other” hospital costs, which included the combined cost of nursing, medication, laboratory, and radiology services.^[32]^ These increased costs can be directly attributed to the increased LOS associated with SSI.^[32,38]^

## 4 Discussion

In an updated systematic review of thirty-four studies, we demonstrate strong evidence of high overall complication incidence arising from colonic resection surgery with associated increased costs and resource utilization. We found a considerable degree of heterogeneity among studies in factors such as study design, defining and reporting on complications, and methodology used to calculate “cost” and associated outcomes. Despite these limitations, our findings confirm that hospital readmissions are associated with significant financial burden, and postoperative complications are associated with greater incidence of hospital readmissions. We found weaker evidence that postoperative SSI and anastomotic leak are associated with greater costs and resource utilization relative to other postoperative complications.

Our review highlights significant shortcomings in defining and reporting of hospital resource utilization in economic studies of postoperative complications in colon resection surgery. First, the measure of hospital resource utilization adopted by the studies varied and was poorly defined in many. Second, the currency year was not reported in most of the studies thus had to be assumed to be the publication year. Thirdly, reporting of costs using means and medians varied, impeding on direct comparison between studies.

Hospital costs, hospital charges and hospital reimbursements are 3 resource use measures that represent different financial aspects of health economics.^[45,46]^ Hospital charges for a given service may differ greatly between hospitals and health care systems and are considered a poor representation of hospital costs.^[47]^ Similarly, hospital reimbursement systems demonstrate significant geographical variation in their coding classification and payment value.^[48]^ In the USA, and many European countries, hospital reimbursements are predetermined and based on DRG codes^[48,49]^ with cost variation within DRG codes acting as a source of uncertainty. As such, the most reliable measure of hospital costs involves recording actual resource consumption for each admission.^[47]^ Secondary to this, studies should clearly define and report the utilized hospital resource use measure to enable accurate analysis of a study's results.

Poorly defined and inconsistent reporting of hospital resources acts as a barrier to accurate comparison of cost and clinical outcomes between studies. Hospital costs consists of fixed direct, variable direct and indirect costs.^[46]^ Inclusion or exclusion of specific hospital cost components resulted in variation in total financial burden of complications amongst the included studies as presented in Figure 3. Furthermore, many studies in our review failed to report the cost currency and currency year which is essential in allowing comparison of cost data. The reporting of health cost studies should adhere to a minimal standard of reporting including the definition of hospital cost components analyzed including any adjustments for inflation that the authors performed. In addition, skewed distributions are expected in medical costing data,^[50]^ therefore it is recommended that both mean and median costs are reported to avoid misinterpretation of results.^[50]^

Most of the included studies reported the cost of a specific complication type improving the clinical relevance of these studies. However, complication definitions were inconsistent across studies limiting the ability to compare complication types. Many studies utilized local institutional definitions or the definitions of the national databases they analyzed. Only seven studies^[19,24,36–38,40,41]^ specified the use of the International Classification of Diseases, Ninth Revision, Clinical Modification (ICD-9-CM) codes to classify complications. Reporting on postsurgical complications should be aligned with established international standards for definitions and use of outcome measures, designed for clinical effectiveness research in perioperative medicine.^[51]^

Additionally, only three studies^[11,18,39]^ assessed the cost impact of complication severity with only one^[39]^ of these utilizing a prevalidated complication severity grading system (the Clavien-Dindo classification system). Our results demonstrated greater hospital resource use with greater complication severity, and as such management and outcomes of complications are heavily reliant on complication severity. Therefore, complication severity is an important factor to analyze and should be reported using internationally validated grading systems such as the Clavien-Dindo classification system.^[52]^

Our review also highlights hospital readmissions as a significant source of costs. This has been recognized internationally. Specifically, in the USA, the hospital readmissions reduction program was introduced which penalizes hospitals who demonstrate high 30-day readmission rates for specific conditions/procedures.^[53]^ This initiative creates a significant financial incentive for hospitals to introduce measures that reduce readmissions. Our review demonstrated increased readmission rates in patients who experience postoperative complications, highlighting the financial benefit of reducing postoperative complication rates. We also recommend that the reporting of readmissions should be standardized to at least 30 days post discharge.

Prevention strategies that aim to mitigate risk factors for complications following colorectal surgery are well described.^[54]^ These include preoperative weight loss,^[54]^ nutritional optimization through immunonutrition^[55]^ and early postoperative enteric nutrition,^[56]^ intraoperative blood loss and blood transfusion minimization,^[57,58]^ and use of laparoscopic surgery if feasible.^[59,60,61]^ Incorporation of these strategies and others into enhanced recovery after surgery management programs has been shown to be associated with reduced postoperative complications,^[60]^ reduced hospital LOS and costs.^[62]^ Despite this, there is a paucity of studies analyzing the cost-benefit outcomes of these strategies. Therefore, large, prospective cost-benefit clinical trials investigating interventions aimed at reducing postoperative complications are still required to improve patient outcomes and the cost-effectiveness of our health care systems.

Our review has several limitations. Notably our review includes a high number of retrospective studies with few high-quality detailed cost outcome studies. Despite an exhaustive search strategy, noninclusion of grey literature may subject our review to a risk of publication bias. In addition, the significant heterogeneity of the included studies prevented a quantitative analysis of the cost results, however the large number of articles identified allowed direct comparison of studies of similar characteristics. High quality prospective economic studies are needed to evaluate the cost of complications arising from colonic resection surgery.

Our systematic review has important clinical implications. We have demonstrated a high prevalence of complications following colon resection surgery and a substantial financial burden associated with complications. Therefore, large, prospective cost-benefit analysis trials investigating strategies aimed at reducing surgical complications and their associated costs are required. Given the significant heterogeneity identified in our review, we propose a standardized approach for future costing studies of postoperative complications. Studies should follow a detailed and consistent methodology with the use of validated economic study guidelines^[63]^ and must report, as a minimum, the following variables: complication definition, complication severity (reported using internationally validated grading systems such as the Clavien-Dindo classification system), follow-up duration for each outcome of interest, mortality reported at 90 days postoperatively and ideally at 1 year in clinical effectiveness research, readmissions standardized to at least 30 days post discharge, hospital cost definition (clearly define and report the utilized hospital resource use measure and the hospital cost components analyzed), cost currency, cost year adjusted for inflation, mean and median cost, confidence intervals and interquartile range as skewed distributions are expected in medical costing data.

## Author contributions

**Conceptualization:** Laurence Weinberg.

**Data curation:** Maleck Louis, Samuel Anthony Johnston, Ronald Ma, Laurence Weinberg.

**Formal analysis:** Maleck Louis, Leonid Churilov, Laurence Weinberg.

**Investigation:** Laurence Weinberg.

**Methodology:** Maleck Louis, Laurence Weinberg.

**Project administration:** Laurence Weinberg.

**Resources:** Samuel Anthony Johnston.

**Supervision:** Christopher Christophi, Laurence Weinberg.

**Validation:** Ronald Ma.

**Writing – original draft:** Maleck Louis, Samuel Anthony Johnston, Leonid Churilov, Ronald Ma, Christopher Christophi, Laurence Weinberg.

**Writing – review & editing:** Maleck Louis, Samuel Anthony Johnston, Leonid Churilov, Ronald Ma, Christopher Christophi, Laurence Weinberg.

## Supplementary Material

Supplemental Digital Content

## Supplementary Material

Supplemental Digital Content

## Supplementary Material

Supplemental Digital Content
