# Supplementary material for: Financial burden of postoperative complications following colonic resection: A systematic review
Source: Medicine (Baltimore). 2021 Jul 9;100(27):e26546. doi: 10.1097/MD.0000000000026546 (PMC8270623; doi:10.1097/MD.0000000000026546)
Supplement: Supplemental Digital Content [file medi-100-e26546-s001.docx]

**Supplementary Table 1 - Search strategy**

| **MEDLINE OVID 19/02/2019** | | |
| --- | --- | --- |
|  | Searches | Results |
| 1 | Exp “Costs and Cost Analysis”/ | 222023 |
| 2 | exp Economics, Hospital/ | 23349 |
| 3 | exp Resource Allocation/ | 16635 |
| 4 | exp Financial Management/ | 85798 |
| 5 | exp Economics, Medical/ | 14076 |
| 6 | (cost-benefit* or cost*benefit* or cost-utili* or cost*utili* or cost-effective* or cost*effective* or health cost* or health expenditure* or health-care cost* or health*care cost* or hospital* cost* or cost-saving* or cost*saving* or cost* analy* or cost comparison* or cost* control* or cost of illness* or cost* allocation* or economic* or pricing or hospital* charge* or (resource* adj3 allocation*) or (resource* adj3 utili*) or (resource* adj3 us*) or hospital financial management).mp. | 810438 |
| 7 | Cost*.tw. | 540664 |
| 8 | 1 or 2 or 3 or 4 or 5 or 6 or 7 | 1116581 |
| 9 | exp Colectomy/ | 19354 |
| 10 | exp Colon/su [Surgery] | 12238 |
| 11 | (Colectom* or hemicolectom* or hartman* procedure or anterior resection* or (sigmoid adj3 resection*) or (sigmoid adj3 surger*) or (large bowel adj3 resection*) or (large intestine adj3 resection*) or (Colon* adj3 resection*) or (colon adj3 surger*) or (large bowel adj3 surger*) or (large intestine adj3 surger*)).mp. | 34836 |
| 12 | 9 or 10 or 11 | 45331 |
| 13 | exp Postoperative Complications/ | 510900 |
| 14 | exp Intraoperative Complications/ | 49816 |
| 15 | (adverse event* or adverse outcome* or complication*).mp. | 3008734 |
| 16 | 13 or 14 or 15 | 3140861 |
| 17 | 8 and 12 and 16 | 936 |
| 18 | limit 17 to yr="2010 -Current" | 562 |
| **EMBASE OVID 19/02/2019** | | |
|  | Searches | Results |
| 1 | Exp economic evaluation/ | 285963 |
| 2 | Exp hospital cost/ | 35733 |
| 3 | Health economics/ | 36605 |
| 4 | exp Resource Management/ | 36520 |
| 5 | Financial Management/ or economic aspect/ | 223919 |
| 6 | “health care cost”/ | 177983 |
| 7 | “cost”/ | 59804 |
| 8 | (cost-benefit* or cost*benefit* or cost-utili* or cost*utili* or cost-effective* or cost*effective* or cost-saving* or cost*saving* or health cost* or health expenditure* or health-care cost* or health*care cost* or hospital* cost* or cost* analy* or cost comparison* or cost* control* or cost of illness* or cost* allocation* or economic* or pricing or hospital* charge* or (resource* adj3 allocation*) or (resource* adj3 utili*) or (resource* adj3 us*) or hospital financial management).mp. | 1081897 |
| 9 | Cost*.tw. | 733541 |
| 10 | 1 or 2 or 3 or 4 or 5 or 6 or 7 or 8 or 9 | 1508792 |
| 11 | exp Colectomy/ | 42109 |
| 12 | (Colectom* or hemicolectom* or hartman* procedure or anterior resection* or (sigmoid adj3 resection*) or (sigmoid adj3 surger*) or (large bowel adj3 resection*) or (large intestine adj3 resection*) or (Colon* adj3 resection*) or (colon adj3 surger*) or (large bowel adj3 surger*) or (large intestine adj3 surger*)).mp. | 63711 |
| 13 | 11 or 12 | 65183 |
| 14 | exp Postoperative Complications/ | 648517 |
| 15 | exp peroperative complication/ | 38714 |
| 16 | (adverse event* or adverse outcome* or complication*).mp. | 3176550 |
| 17 | 14 or 15 or 16 | 3326785 |
| 18 | 10 and 13 and 17 | 1778 |
| 19 | limit 18 to yr="2010 -Current" | 1267 |
| **Cochrane Library (Wiley Online Library) 19/02/2019** | | |
|  | Searches | Results |
| 1 | MeSH descriptor: [Costs and Cost Analysis] explode all trees | 9580 |
| 2 | MeSH descriptor: [Health Care Economics and Organizations] explode all trees | 19188 |
| 3 | MeSH descriptor: [Financial Management] explode all trees | 239 |
| 4 | health expenditure* or economic* or pricing or hospital* charge* or (resource* near/3 allocation*) or (resource* near/3 utili*) or (resource* near/3 us*) or hospital financial management or cost* | 69058 |
| 5 | #1 or #2 or #3 or #4 | 76934 |
| 6 | MeSH descriptor: [Postoperative Complications] explode all trees | 35638 |
| 7 | MeSH descriptor: [Intraoperative Complications] explode all trees | 4002 |
| 8 | adverse event* or adverse outcome* or complication* | 248772 |
| 9 | #6 or #7 or #8 | 261378 |
| 10 | MeSH descriptor: [Colectomy] explode all trees | 666 |
| 11 | MeSH descriptor: [Colon] explode all trees and with qualifier(s): [surgery - SU] | 555 |
| 12 | MeSH descriptor: [Colorectal Surgery] explode all trees | 171 |
| 13 | Colectom* or hemicolectom* or hartman* procedure or anterior resection* or (sigmoid near/3 resection*) or (sigmoid near/3 surger*) or (large bowel near/3 resection*) or (large intestine near/3 resection*) or (Colon* near/3 resection*) or (colon near/3 surger*) or (large bowel near/3 surger*) or (large intestine near/3 surger*) | 3720 |
| 14 | #10 or #11 or #12 or #13 | 4234 |
| 15 | #5 and #9 and #14  Limit: Cochrane Library publication date from Jan 2010 to Feb 2019 | 455 |
| **EconLit (EBSCOhost) 19/02/2019** | | |
|  | Searches | Results |
| 1 | surg* or operation or operat* or surgical procedure or resection or lap* | 84171 |
| 2 | complication* or adverse or mortality or death | 23996 |
| 3 | colo* resection* or colectomy* or colo* surger* or large bowel or large intestine or colon or colonic* | 85 |
| 4 | 1 and 2 and 3 | 6 |
| 5 | Limit to publication date 2010-2019 | 5 |
