# Supplementary material for: Financial burden of postoperative complications following colonic resection: A systematic review
Source: Medicine (Baltimore). 2021 Jul 9;100(27):e26546. doi: 10.1097/MD.0000000000026546 (PMC8270623; doi:10.1097/MD.0000000000026546)
Supplement: Supplemental Digital Content [file medi-100-e26546-s002.docx]

**Supplementary Table 2 - Summary and results of primary outcomes of included studies reporting on resource use/costs of postoperative complications following colonic resection surgery.**

| **Author, year** | **Study design** | **Country, currency and cost year** | **Patients (n) and dates of patient sample** | **Intervention** | **Follow up period post-operatively and outcome definition** | **Incidence of complications** | **Cost of complications** |
| --- | --- | --- | --- | --- | --- | --- | --- |
| **Studies reporting on hospital costs** | | | | | | | |
| **Braga et al., 2010** | Single-centre randomised clinical trial | Italy,  Euro (€) assumed 2010 | 268 patients undergoing elective left colon resection;  February 2000 – December 2004 | Open left colonic resection =134 (50%)  Laparoscopic left colonic resection =134 (50%) | Costs:  Hospital costs including operative costs, admission costs and costs 30-day post discharge.  Postoperative complications  Patients categorised based on presence or absence of complications. Complications identified based on a priori definition.  *Complication severity not reported*    *Postoperative follow-up period is 30-days post discharge* | Postoperative complication incidence  Laparoscopic colon resection =16 (11.9%)  Open colon resection = 27 (20.1%)  ; P-value = 0.094 | Mean additional cost of complications  Laparoscopic colonic resection = €1 478.63 **[$2 290]**  Open colonic resection = €2 662.89 **[$4 123]**  *Cost of complication calculated by dividing the total cost of complications in the laparoscopic and open groups by the number of patients experiencing a complication within each group respectively*  Cost breakdown not available |
| **Sammour et al., 2010** | Single-centre retrospective cohort study  ERAS group was collected prospectively  Control group was collected retrospectively | New Zealand,  NZD ($) assumed 2010 | 100 patients undergoing elective colonic surgery;  50 consecutive patients enrolled in the ERAS programme between  December 2005 and March 2007  50 consecutive patients prior to commencement of ERAS programme between September 2004 – September 2005 | Open and laparoscopic colon resection techniques included and analysed as *one group* | Costs:  Hospital costs defined as cost of index hospital stay excluding cost of day stay and readmissions.  Postoperative complications  Patients categorised based on presence or absence of post-operative complications and by complication type.  *Complication severity not reported*  *Postoperative follow-up period is 30-days post operatively* | Incidence of post-operative complications  ERAS group =27 (54.0%)  Control Group =33 (66.0%)  ; P-value=0.221  Breakdown by complication type reported | Incremental cost of complication by complication type  Leak / collection = $34 853.26 **[$29 340]**  Ileus = $6 517.37 **[$5 486]**  Wound complication = $19 703.81 **[$16 587]**  Urinary tract infection = $4 615.13 **[$3 885]**  Urinary retention = $3 445.41 **[$2 900]**  Cardiopulmonary = $10 802.13 **[$9 093]**  Weighted mean additional cost  Any complication = $ $20 278 **[$17 070]**  Cost breakdown not available |
| **Bloom et al., 2011** | Multi-centre retrospective cohort study utilising The Premier Research Database  *(Conference abstract)* | USA;  USD ($) assumed 2011 | 132 231 patients out of which 34 295 patients underwent colectomy,  2009 | Laparoscopic colectomy = 11 366 (33.1%)  Open colectomy = 22 929 (66.9%) | Costs:  Total hospitalisation cost. Definition not specified  Postoperative complications  Patients categorised based on the presence or absence of postoperative pulmonary complications (PPC)  *Complication severity not reported*  *Follow-up period not reported* | Laparoscopic colectomy  PPC incidence rate 9.4%  Open colectomy  PPC incidence rate 26.3% | Mean total cost of PPC for laparoscopic colectomy  Patients with PPC = $28 759 **[$33 010]**  Patients without PPC= $24 013 **[$27 563]**  ; P-value<0.0001  Mean total cost of PPC for open colectomy  With PPC = $34 053 **[$39 087]**  Without PPC = $25 524 **[$29 297]**  ; P-value <0.0001  Cost breakdown not available |
| **Delissovoy et al., 2011** | Multi-centre retrospective cohort study utilising the Premier Perspective™ Comparative Database  *(Conference abstract)* | USA;  USD ($) assumed 2011 | Number of patients undergoing colon procedures not reported;  2007-2010 | Colon resection;  Surgical technique not specified | Costs:  Hospital costs. Definition not reported  Postoperative complications  Patients categorised based on the presence or absence of SSI  *Complication severity not reported*  *Follow-up period not reported* | Incidence of SSI = 12.0%, (95%CI: 11.78-12.2%) | Mean (95%CI) additional cost of SSI = $19 349 ($19 315-$19 383) **[$22 209]**  No P-value  Cost breakdown not reported |
| **Ramamoorthy, 2012** | Multi-centre retrospective cohort study utilising a national database (unspecified)  *(Conference abstract)* | USA,  USD ($) assumed 2012 | 12 620 patients undergoing laparoscopic colectomy;  September 2008 – September 2010 | Laparoscopic colectomy | Costs:  Hospital costs. Definition not given  Postoperative complications  Patients categorised based on the presence or absence of opioid-related adverse events (ORAE)  *Complication severity not reported*  *Postoperative follow-up period not specified* | Incidence of ORAE not reported | Mean total costs  Patients with ORAE = $18 322 **[$20 433]**  Patients without ORAE= $15 720 **[$17 531]**  ; P-value <0.0001  *Cost breakdown not available* |
| **Kashimura et al, 2012** | Multi-centre retrospective-matched cohort study | Japan;  USD ($) assumed 2012  Converted from Yens (¥1 = US$0.01) and Euro (€1 = US$1.5) | 334 patients out of which 204 patients underwent colon resection;  April 2006 – March 2008 | open colon resection =154 (75.5%)  laparoscopic colon resection =50 (24.5%) | Costs:  Hospital costs, defined as cost of index admission post-surgery, plus any costs incurred during readmission secondary to SSI. Costs were calculated by using the fee-for-service calculation method.  Postoperative complications  Patients were categorised based on presence or absence of SSI. SSI were identified using Centers for Disease Control and prevention criteria.  *Complication severity not reported for colon resection patients specifically*  *Postoperative follow-up period was defined as 30 days after surgery* | No Data  Patients with and without SSI were case-matched  With SSI =102  Without SSI =102 | Mean cost (SD) for entire cohort  With SSI = $10 152 (13 474) **[$11 322]**  Without SSI = $4 279 (2 945) **[$4 772]**  Mean additional cost of SSI = $5 873 [95%CI: 3 166 to 8 579] **[$6 550]**  ; P-value <0.001  *Cost breakdown not available* |
| **Kalogera et al., 2013** | Single-centre retrospective cohort study  *(Conference abstract)* | USA,  USD ($) assumed 2013 | 42 Anastomotic leak cases matched with 84 no-leak controls undergoing large bowel resection for primary ovarian cancer;  1994 - 2011 | Large bowel resection;  surgical technique not specified | Costs:  Hospital costs at 30 days, 3 months, 6 months and 1 year excluding outpatient cost data.  Postoperative complications  Patients categorised based on presence or absence of anastomotic leak.  *Complication severity not reported*  *Postoperative follow-up period is 1-year post operatively* | Case-matched population with 42 patients with anastomotic leaks | Median (IQR) cost at 30 days  Patients with anastomotic leak = $72 760.4 (52 858.9-104 449.2) **[$79 868]**  Patients without anastomotic leaks = $33 453.7 (27 081.0-41 743.0) **[$36 722]**  ; P-value<0.0001  *Cost breakdown not available* |
| **Thacker et al., 2014** | Multi-centre retrospective cohort study utilising the Premier research database (U.S)  *(Conference abstract)* | United Kingdom;  USD ($) assumed 2014 | 84 722 patients undergoing elective colon surgery;  January 2008 – June 2012 | Colon surgery;  Surgical technique not specified. | Costs:  Hospital costs. Definition not reported  Postoperative complications  Patients categorised into those with and without POI identified using ICD-9-CM codes.  *Complication severity not reported*  *Postoperative follow-up period not specified.* | Incidence of POI =14 972 (17.7%) | Mean (±SD) total hospital costs  With POI= $20 734 (±14 506) **[$22 406]**  Without POI = $13 865 (±8 315) **[$14 983]**  ; P-value <0.0001  *Cost breakdown not available* |
| **Asgeirsson et al., 2014** | Single-centre retrospective cohort study | USA;  USD ($) assumed 2014 | 1 422 patients undergoing segmental colectomy;  July 2008 – June 2012 | Laparoscopic segmental colectomy = 654 (46.0%)  Open Segmental colectomy = 768 (54.0%) | Costs:  Hospital costs derived from the institutional cost accounting system for 30 days post-surgery  Postoperative complications:  Patients categorised based on type of complication as well as number of complications experienced (0,1,2,3+).  Complications with incidence <3% were excluded from analysis.  *Postoperative follow-up period is 30 days post-surgery* | Complication incidence in the laparoscopic group:  No complication = 569 (87.0%)  1 complication = 44 (6.7%)  2 complications = 20 (3.1%)  3+ complications = 21 (3.2%)  Complication incidence in the open group:  No complication = 461 (60.0%)  1 complication = 114 (14.8%)  2 complications = 70 (9.1%)  3+ complications = 123 (16.0%)  Complication incidence by complication type:  Post-operative Ileus =26/1 422  Postoperative anaemia =58/1 422  Surgical Site Infection =12/1 422  Deep Surgical Site infection/Organ space (including anastomotic leak) =27/1 422  Unplanned return to the operative room =24/1 422 | Mean (± SD) cost in laparoscopic group:  No complication = $7 739 (± 4 150) **[$8 363]**  1 complication = $11 878 (± 7 266) **[$12 836]**  2 complications = $12 465 (± 4 153) **[$13 470]**  3+ complications = $24 549 (± 11 816) **[$26 528]**  *Weighted mean additional cost*  *Any complication = $7 408* ***[$8 005]***  Mean (± SD) cost in open group:  No complication = $10 935 (± 12 498) **[$11 817]**  1 complication = $13 989 (± 9 570) **[$15 117]**  2 complications = $18 300 (± 11 218) **[$19 776]**  3+ complications = $30 529 (± 21 704) **[$32 991]**  *Weighted mean additional cost*  *Any complication = $10 664* ***[$11 524]***  Mean (± SD) cost by complication type in overall patient sample:  Postoperative Ileus = $14 529 (± 12 953) **[$15 700]**  Postoperative anaemia = $12 757 (± 8 644) **[$13 786]**  Surgical Site Infection = $31 899 (± 14 705) **[$34 471]**  Deep SSI/organ space (including anastomotic leak) = $39 902 (± 27 911) **[$43 119]**  Unplanned return to operating room = $26 536 (± 20 841) **[$28 676]**  No P-values  *Cost breakdown not available* |
| **Flynn et al., 2014** | Single-centre retrospective cohort study | USA;  USD ($) 2010 | 276 patients undergoing open partial colectomy with anastomosis;  2007 - 2010 | All patients underwent open partial colectomy with anastomosis | Costs:  Hospital costs defined as cost of index admission, plus any costs incurred during readmission within 30 days. Costs included total costs, fixed costs and variable costs.  Estimated reimbursement and total margin (profitability) were also reported.  Postoperative complications:  Patients categorised based on presence or absence of major complications. The standard  ACS-NSQIP definitions were used to identify complications.  *Complication severity not reported*  *Postoperative follow-up period is 30 days post-surgery* | Complication incidence  61 (22.1%) patients experienced one or more major complication  Complication incidence by complication type:  Deep SSI = 5 (1.8%)  Organ/space SSI = 14 (5.1%)  Sepsis = 5 (1.8%)  Clostridium difficile colitis = 4 (1.4%)  Pneumonia = 8 (2.9%)  Bowel obstruction =11 (4.0%)  Acute kidney injury = 6 (2.2%)  Cerebrovascular accident = 2 (0.7%)  Pulmonary embolism = 4 (1.4%)  New-onset arrhythmia = 13 (4.7%)  Myocardial infarction = 5 (1.8%)  Cardiac arrest = 1 (0.4%)  Unplanned intubation = 6 (2.2%)  Reoperation = 13 (4.7%)  Death = 1 (0.4%)  Other infections = 7 (2.5%) | Mean (± SD) total cost for entire cohort (n=276)  No complication = $23 101 (±6 914) [**$26 948]**  1+ complication = $48 180 (±26 596) **[$56 204]**  Difference = $25 079 **[$29 256]**  ; P-value <0.001  Mean (± SD) total cost for matched sample (N=100; 50 complicated and 50 uncomplicated)  No complication = $23 381 (±6 712) **[$27 275]**  1+ complication = $49 733 (±28 279) **[$58 016]**  Difference = $26 351 **[$30 740]**  ; P-value <0.001    *Cost breakdown not available* |
| **Manecke et al., 2014** | Multi-centre retrospective cohort study utilising the University Health system Consortium database | USA,  USD ($) assumed 2014 | 75 140 patients out of which 19 055 patients underwent a colonic resection;  2011 | Open and laparoscopic colon resection techniques included and analysed as *one group* | Costs:  Direct hospital costs excluding health care personnel wages and overhead costs.  Postoperative complications  Patients categorised based on presence or absence of postoperative complications. The University Health system Consortium definitions were used to identify postoperative complications  *Complication severity not reported*  *Postoperative follow-up period is defined as the index hospital admission* | Complication incidence = 16.0% | Mean (± SD) Direct cost  1+ complication = $49 160 (± 56 975) **[$53 124]**  No complication = $17 158 (± 16 481) **[$18 541]**  ; P-value <0.001    *Cost breakdown not available* |
| **Gan et al., 2015** | Multi-centre retrospective cohort study utilising The Premier Research Database | USA;  USD ($) assumed 2015 | 138 068 patients out of which 57 948 patients were undergoing colon resection;  September 2008 – August 2010 | Open colon resection =40 250 (69.5%)  Laparoscopic colon resection =17 698 (30.5%) | Costs:  Hospital costs derived from all billed items at the individual patient level. This includes categories such as medications, operative procedures, diagnostic procedures, laboratory tests and professional and administrative costs.  Postoperative complications  Patients categorised based on presence or absence of POI identified using ICD-9-CM codes  *Complication severity not reported*  *Postoperative follow-up period is unclear – readmission followed up till 30 days post discharge* | Incidence of postoperative ileus  Laparoscopic resection = 2577 (14.6%)  Open resection = 8303 (20.6%) | Median cost for Laparoscopic colon resection:  With POI = $17 505 **[$18 933]**  Without POI = $12 521 **[$13 543]**  ; P-value <0.0001  Median cost for open colon resection:  With POI = $24 078 **[$26 043]**  Without POI = $17 044 **[$18 435]**  ; P-value <0.0001  *Cost breakdown not available* |
| **Knechtle et al., 2015** | Single-centre retrospective cohort study utilising the ACS-NSQIP site specific database | USA;  USD ($) assumed 2015 | 859 patients out of which 390 patients underwent a colon resection;  August 2009 – September 2012 | Open and laparoscopic colon resection techniques included and analysed as *one group* | Costs:  Hospital cost defined as cost of index admission, plus any costs incurred during readmission within 30 days, excluding professional fees.  Total charges, best payment estimate, contribution margin and net profit and also reported.  Postoperative complications  Patients categorised base on presence or absence of postoperative surgical complications and by number of complications experienced. The  ACS-NSQIP definitions were used to identify postoperative surgical complications  Complication severity not reported  *Postoperative follow up period was 30 days post discharge* | Postoperative surgical complication incidence  1+ complication =126 (32.3%)  0 complication =264 (67.7%)  1 complication =74 (19.0%)  2 complications =25 (6.4%)  3 complications =11 (2.8%)  4+complications =16 (4.1%) | Average total cost for entire cohort:  0 complications =$25 688.89 **[$27 785]**  1 complication =$41 942.32 **[$45 364]**  2 complications =$56 641.12 **[$61 262]**  3 complications =$79 539.73 **[$86 029]**  4+ complications =$95 119.25 **[$102 880]**  Trend with increasing number of complications is statistically significant (P-value <0.0001)  Total cost increased by a factor of 1.25 for each additional complication, adjusting for confounders (e^β^: 1.25, P-value < 0.001).  Weighted mean additional cost  Any complication = $29 294.8 **[$31 588]**  *Cost breakdown not available* |
| **Phothong et al., 2015** | Single-centre retrospective cohort study | Thailand;  USD ($)  assumed 2015 | 100 case-matched patients undergoing sigmoidectomy;  January 2008 – September 2013 | HALS sigmoidectomy =50 (50%)  Open sigmoidectomy =50 (50%) | Costs  Hospital costs including room charges, operating room costs, anaesthesia costs, instrument costs, other hospital costs (nursing, medication, laboratory, and radiology), and total costs.  Postoperative complications  Patients categorised based on presence or absence of complications and type of complication: anastomosis leakage, bowel ileus, surgical site infection (SSI) and lung complication. Complication definition not reported.  *Complication severity not reported*  *Postoperative follow-up period is unclear* | Complication incidence by complication type:  Anastomosis leakage = 2 (2%)  Bowel ileus = 5 (5%)  SSI = 6 (6%)  Lung complication = 2 (2%) | Cost data available for SSI in open surgery group only.  Median (range) hospital cost  Total hospital costs:  With SSI = $4 348 ($2 185-$11 509) **[$4 703]**  Without out SSI = $2 140 ($1 379-$6 277) **[$2 315]**  ; P-value=0.004  Room costs:  With SSI = $466 ($156-$661) **[$504]**  Without SSI = $184 ($55-$1 413) **[$199]**  ; P-value=0.024  Operative costs:  With SSI = $823 ($432-$1 278) **[$890]**  Without SSI = $543 ($260-$1 176) **[$587]**  ; P-value=0.020  Anaesthesia costs:  With SSI = $267 ($145-$490) **[$289]**  Without SSI = $225 ($101-$407) **[$243]**  ; P-value = 0.107  Instrument costs:  With SSI = $237 ($19-$852) **[$256]**  Without SSI = $127 ($0-$951) **[$137]**  ; P-value = 0.257  Other hospital costs:  With SSI = $1 966 ($569-$7 991) **[$2 126]**  Without SSI = $569 ($353-$3 623) **[$615]**  ; P-value= 0.003 |
| **Manzanares et al., 2016** | Single-centre randomised control trial | Spain;  Euro (€) assumed 2016 | 84 patients out of which 46 patients underwent Colon resection;  December 2010 – October 2011 | Colon resection;  surgical technique not specified | Costs:  Hospital costs. Determined by multiplying the daily DRG assigned cost by total length of stay + cost of preoperative oral supplements.  Postoperative complications  Patients categorised based on presence or absence of post-operative infections which were further classified as minor (surgical wound infection, phlebitis, bacteraemia, urinary tract infection) and major (anastomotic leak) post-surgical infections.  *Complication severity not reported for costing analysis*  *Postoperative follow-up period is 30-days post discharge* | Incidence of post-operative infectious complications  Overall infectious complications =18 (39.1%)  Patients with minor complications =16 (34.8%)  Patients with major complications =5 (10.9%) | Mean (±SD) reimbursements  Patients with infectious complications = €10 663.5 (±5 680.6) **[$12 590]**  Patients without infectious complications = €5 131.4 (± 3 539) **[$6 058]**  ; P-value <0.0001  Cost breakdown not available |
| **Healy et al., 2016** | Single-centre retrospective cohort study utilising the Michigan Surgical  Quality Collaborative and internal cost accounting data  available at the University of Michigan Health System. | USA;  USD ($) assumed 2016 | 5 120 surgical patients, number of patients undergoing colectomy not reported;  January 2008 – April 2015 | Open and laparoscopic colon resection techniques included and analysed as *one group* | Costs:  Hospital costs of index admission episode excluding professional physician fees. Categorised into fixed, variable, direct and indirect costs.  Reimbursement, out-of-pocket expenses and profit margin also reported.  Postoperative complications  Patients categorised based on presence or absence of complications for colectomy. Complications were identified based on Michigan Surgical Quality Collaborative criteria.  *Complication severity not reported*  *Postoperative follow-up period is not specified* | Incidence of complications for colon resection not reported | Total hospital costs  With complication = $41 135 **[$43 889]**  Without complication = $23 285 **[$24 844]**  Hospital reimbursements  With complications = $42 675 **[$45 532]**  Without complications = $27 285 **[$29 111]**  Profit margin (profit divided by reimbursement)  With complications = 10%  Without complications = 18%  Cost breakdown not available |
| **Zogg et al., 2016** | Multi-centre retrospective cohort study utilising the Nationwide Inpatient Sample | USA;  USD ($) 2014 | 68 462 patients undergoing elective colon resection weighted to represent 337 887 patients nationally;  2009-2011 | Laparoscopic colon resection =35 068 (51.2%)  Open and other colon resection =33 395 (48.8%) | Costs  Direct hospital costs of index hospitalisation calculated by conversion of hospital charges to total hospital costs using the Healthcare Cost and Utilization Project cost-to-charge ratio files.  Postoperative complications  Patients were categorised based on absence or presence of complication and specified system-based complication groups identified using ICD-9-CM codes.  *Complication severity not reported*  *Postoperative follow-up period is not specified* | Complication incidence by complication type  Any complication= 55 549 (16.4%)  Mechanical wound =3 379 (1.0%)  Infection = 13 178 (3.9%)  Urinary complication =3 717 (1.1%)  Pulmonary complication = 6 420 (1.9%)  Gastrointestinal tract complication =29 732 (8.8%)  Cardiovascular complication =6 082 (1.8%)  Systemic complication =1 014 (0.3%)  Complications during surgical procedures =5 406 (1.6%) | Risk-adjusted predicted mean (95%CI) incremental costs of any complication by principal diagnosis in open colon resection  Colon cancer =$11 038.36 (10 536–11 539) **[$11 928]**  Diverticular disease =$9 515.66 ($8 883–10 147) **[$10 283]**  Benign colonic neoplasm =$9 146.55 ($8 307– 9 985) **[$9 884]**  Regional enteritis/Ulcerative colitis =$10 597.33 ($9 200–12 000) **[$11 452]**  Risk-adjusted predicted Mean (95%CI) incremental costs of any complication by principal diagnosis in laparoscopic colon resection  Colon cancer =$8 872.08 ($8 320–9 423) **[$9 587]**  Diverticular disease =$7 219.52 ($6 768–7 670) **[$7 802]**  Benign colonic neoplasm =$7 931.67 ($7 413–8 450) **[$8 571]**  Regional enteritis/Ulcerative =$7 685.56 ($6 202–9 169) **[$8 305]**  *Risk adjusted predicted mean incremental costs (95% CI) stratified by complication type reported*  *No P-value reported*  *Cost breakdown not available* |
| **Martin et al., 2017** | Multi-centre retrospective cohort study utilising the national Veterans Affairs Surgical Quality Improvement Program and the national VA Corporate Data Warehouse  *(Conference abstract)* | USA;  USD ($) assumed 2017 | 7 102 Veterans undergoing elective colectomy for benign polyps;  2000 - 2014 | Laparoscopic colectomy =2 417 (34.0%)  Open colectomy =4 685 (66.0%) | Costs:  Hospital costs. Definition not reported  Postoperative complications  Patients categorised based on presence or absence of complications. This study included mortality as a complication.  *Complication severity not reported for costing analysis*  *Postoperative follow-up period not specified.* | Overall complication rate =1 398 (19.7%)  Complication incidence by complication type  Cardiac arrest = 51 (0.7%)  CVA/Stroke =10 (0.1%)  DVT/Thrombophlebitis =34 (0.5%)  Sepsis = 189 (2.7%)  Pneumonia = 195 (2.8%)  Pulmonary Embolism = 40 (0.6%)  Reintubation = 170 (2.4%)  Acute renal failure = 43 (0.6%)  Renal insufficiency = 62 (0.9%)  UTI = 186 (2.6%)  Deep wound SSI = 61 (0.9%)  Superficial SSI = 538 (7.6%)  Wound dehiscence = 145 (2.0%) | Median (IQR) cost of complications in open colon resection  Without complication = $22 712 (IQR: $16 621-32 508) **[$23 641]**  With complication = $32 460 (IQR: $20 842-54 665) **[$33 788]**  Median (IQR) cost of complications in laparoscopic colon resection  Without complication =$20 697 (IQR: $14 905-29 268) **[$21 544]**  With complication = $27 639 (IQR: $19 752-45 748) **[$28 770]**  *No P-value*  *Cost breakdown not reported* |
| **Zogg et al., 2018** | Multi-centre retrospective cohort study utilising the Nationwide Inpatient Sample | USA;  USD ($) 2017 | 293 967 patients out of which 217 939 patients underwent a colonic resection;  2001 – 2014  Modelling used Nationwide Inpatient Sample-provided population weights to extrapolate the sample to a nationally representative version of the US population | Open and laparoscopic colon resection techniques included and analysed as *one group* | Costs:  Direct hospital costs of index hospitalisation – total hospital charges converted to total hospital costs using cost-to-charge ratios.  Postoperative complications  Patients were categorised based on absence or presence of complication and further categorised based on specified system-based complication groups identified using ICD-9-CM codes  *Complication severity not reported*  *Postoperative follow-up period is not specified* | Risk adjusted probability (95%CI) of complications by complication type  Any complication – 24.4% (24.0–4.8%)  Mechanical wound - 1.7% (1.7–1.8%)  Infection 5.9% (5.8–6.1%)  Urinary - 1.4% (1.3–1.4%)  Pulmonary - 3.7% (3.6–3.9%)  Gastrointestinal - 12.0% (11.7–12.3%)  Cardiovascular - 3.0% (2.9–3.1%)  Systemic - 1.0% (1.0–1.1%)  Surgical - 3.1% (3.0–3.2%) | Risk-adjusted predicted mean (95%CI) additional cost by complication type  Base value (No complication) =$19 134.92 ($18 900–19 400) **[$19 918]**  Any complication =$15 486.34 ($15 100–15 900) **[$16 120]**  ; P-value < 0.001  Mechanical wound =$11 810.92 ($11 100–12 500) **[$12 294]**  ; P-value < 0.001  Infection =$17 452.93 ($17 000–17 900) **[$18 167]**  ; P-value < 0.001  Urinary - $3 124.35 ($2 400–3 800) **[$3 252]**  ; P-value < 0.001  Pulmonary - $11 890.64 ($11 400–12 400) **[$12 377]**  ; P-value < 0.001  Gastrointestinal - $5 958.92 ($5 700–6 300) **[$6 203]**  ; P-value < 0.001  Cardiovascular - $7 778.60 ($7 300–8 300) **[$8 097]**  ; P-value < 0.001  Systemic - $4 699.68 ($3 800–5 600) **[$4 892]**  ; P-value < 0.001  Surgical - $6 823.41 ($6 300–7 300) **[$7 103]**  ; P-value < 0.001  *Cost breakdown not available* |
| **Studies reporting on hospital charges** | | | | | | | |
| **Fukuda et al., 2012** | Multi-centre retrospective cohort study utilising the Diagnosis Procedure Combination/  Per-Diem Payment System and  Japan Nosocomial Infections Surveillance databases | Japan;  USD ($) 2010  Converted and adjusted from Yens (US$1 = ¥122.1) | 1 817 patients out of which 1 308 patients were undergoing colon resection;  September 2007 to December 2010  *Only 1 108 colon resection patients had data for postoperative resource consumption* | Laparoscopic colon resection = 381 (29.1%)  Open colon resection = 927 (70.9%) | Costs:  Hospital charges defined as expenditure incurred between the first day postoperatively and the day of discharge (surgery charges on the day of operation were not included). Charges were calculated by multiplying the volume of resources consumed per patient by the official unit price  Postoperative complications:  Patients categorised based on presence or absence of SSIs. Severity of SSI reported as superficial, deep and space/organ SSI.  The standard Centres for Disease Control and Prevention National Nosocomial Infections Surveillance System criteria were used for identifying SSIs.  *Postoperative period is defined as interval between first postoperative day and discharge day.* | Overall SSI incidence = 156/1 308 (11.9%)  Complication incidence by SSI severity:  Superficial surgical site infection =73/1 108 (6.6%)  Deep surgical site infection =15/1 108 (1.4%)  Space/organ surgical site infection =9/1 108 (0.8%) | Mean (95% CI) postoperative charge for overall patient sample:  With SSI = $4 189 (4 114 – 4 266) **[$4 887]**  Without SSI = $2 973 (2 919–3 033) **[$3 468]**  Difference = $1 216 (1 196–1 240) **[$1 419]**  ; P-value <0.001  Mean (95% CI) additional postoperative charge for open surgery  With superficial SSI = $853 (834–873) **[$995]**  ; P-value = 0.002  With deep SSI = $1 847 (1 808–1 886) **[$2 155]**  ; P-value = 0.024  With space/organ SSI = $2 003 (1 957–2 046) **[$2 337]**  ; P-value<0.001  Mean (95% CI) increase in postoperative charge for Laparoscopic surgery  With superficial SSI = $739 (717–763) **[$862]**  ; P-value<0.001  With deep SSI = $1 672 (1 619– 1 726) **[$1 950]**  ; P-value = 0.016  No patient developed space/organ infection in laparoscopic colon surgery.  *Cost breakdown not available* |
| **Vaid et al., 2012** | Multi-centre retrospective cohort study utilising the 2008 Cost &  Utilization Project National Inpatient Sample | USA;  USD ($) assumed 2012 | 63 950 patients undergoing elective colonic resection;  2008 | Laparoscopic colon resection =5 147 (8.0%)  Open colon resection = 58 802 (92%) | Costs:  Total charges – definition not given.  Postoperative complications  Patients were categorised based on absence or presence of complications divided into system-based complication groups. Complications were identified using ICD-9-CM Codes.  *Complication severity not reported*  *Postoperative follow-up period is not specified* | Overall complication incidence = 16 865 (26.4%)  Complication incidence by surgical technique  Laparoscopic = 974 (18.9%)  Open = 15 891 (27.1%)  ; P-value <0.001  *Incidence of system-based complication groups reported.* | Additional total hospital charge of complication was $37 264 **[$41 557]** for combined open and laparoscopic  Median charge in laparoscopic colon resection:  Without complication =$39 030 **[$43 526]**  With complication =$58 382 **[$65 108]**  ; P-value < 0.001  Median charge in open colon resection  Without complication =$39 152 **[$43 662]**  With complication =$62 221 **[$69 389]**  ; P-value < 0.001  *Cost breakdown not available* |
| **Studies reporting on hospital reimbursements** | | | | | | | |
| **Birkmeyer et al., 2010** | Multi-centre retrospective cohort study utilising Medicare claims data | USA,  USD ($)  Assumed 2010 | 7 181 patients undergoing colectomy for colon cancer;  2005 | Colectomy;  Surgical technique not specified | Costs:  Hospital reimbursements defined as payments to hospitals for index admission and 30 days post discharge.  Postoperative complications  30-day readmission  *Postoperative follow-up period is 30-days post discharge* | Incidence of complications for colon resection not reported | No Data  See Supplementary Table 3 for cost of readmission |
| **Wick et al., 2011** | Multi-centre retrospective cohort study utilising Blue Cross and Blue Shield insurance plan claims database | USA,  USD ($) assumed 2011 | 7 020 patients undergoing partial or total colectomy;  January 2002 – December 2008 | laparoscopic colon resection = 1 273 (18.1%)  Open colon resection = 5 747 (81.9%)  Open and laparoscopic colon resection techniques analysed as *one group* in costing analysis | Costs:  Hospital reimbursements defined as payments to hospitals from day of operation to 90 days postoperatively including total hospital, emergency department, home health, and outpatient pharmacy services.  Postoperative complications  Patients categorised based on presence or absence of surgical site infection within 30 days after the operation. Postoperative infections were identified using ICD-9-CM codes.  *Complication severity not reported*  *Postoperative follow-up period is 90-days postoperatively* | Overall incidence of SSI =726 (10.3%) | Postoperative SSI increased the reimbursement for colectomy by $17 324 **[$19 886]** in open and laparoscopic colon resection combined.  Mean (95%CI) reimbursement for SSI by cost category:  **Total cost**  With SSI = $31 933 (29 607-34 258) **[$36 653]**  Without SSI = $14 608 (14 018-15 197) **[$16 767]**  ; P-value <0.001  **Inpatient costs**  With SSI = $26 307 (24 045-28 569) **[$30 196]**  Without SSI = $11 029 (10 488-11 507) **[$12 659]**  **Ambulatory costs**  With SSI = $4 174 (3 617-4 730) **[$4 791]**  Without SSI = $3 120 (2 934-3 305) **[$3 581]**  **Emergency department costs**  With SSI = $587 (416-759) **[$674]**  Without SSI = $184 (148-220) **[$211]**  **Home care costs**  With SSI = $1 294 (1 062-1 526) **[$1 485]**  Without SSI = $253 (225-280) **[$290]**  **Pharmacy costs**  With SSI = $699 (575-824) **[$802]**  Without SSI = $463 (435-492) **[$531]** |
| **Birkmeyer et al., 2012** | Multi-centre retrospective cohort study utilising Medicare claims data | USA;  USD ($)  assumed 2012 | 73 772 patients undergoing colon resection for colon cancer at 1 227 hospitals;  January 2005 – November 2007  Hospitals categorised into risk quintiles based on mortality and complication rates | Colectomy;  Surgical technique not specified | Costs:  Hospital reimbursements defined as payments to hospitals for index admission and 30 days post discharge. Payments were categorised into index  hospitalization, readmissions, physician services, and post-discharge ancillary care costs.  Postoperative complications  30-day readmission  Patients categorised based on presence or absence of complications identified using the Complication Screening Project codes  *Postoperative follow-up period is 30-days post discharge* | Complication incidence by hospital risk quintile  Lowest-risk hospitals = 12.6%  Highest-risk hospitals = 28.8% | No Data  See Supplementary Table 3 for cost of readmission |
| **Regenbogen et al., 2012** | Multi-centre retrospective cohort study utilising Medicare claims data | USA;  USD ($)  assumed 2012 | 101 349 patients undergoing colectomy for colon cancer;  January 2005 – November 2007  Hospitals categorised into risk quintiles based on procedure volumes | Colectomy;  Surgical technique not specified | Costs:  Hospital reimbursements defined as payments to hospitals for index admission and 30 days post discharge.  Postoperative complications  30-day readmission.  Patients categorised based on presence or absence of complications identified using the Complication Screening Project codes  *Postoperative follow-up period is 30-days post discharge* | Complication incidence by hospital volume quintile  Low-volume hospital = 20.4%  High-volume hospital = 19.6% | No Data  See Supplementary Table 3 for cost of readmission |
| **Lawson et al., 2013** | Multi-centre retrospective cohort study utilising the ACS-NSQIP and Medicare inpatient claims data  *(Conference abstract)* | USA;  USD ($) assumed 2013 | 90 932 patients out of which 13 644 patients underwent a colectomy;  2005 - 2008 | Colectomy;  Surgical technique not specified | Costs:  Hospital reimbursements defined as Medicare payments to hospitals for 30-day postoperative readmissions  Postoperative complications  30-day readmission rates in patients with and without 30-day postoperative complication based on ACS-NSQIP definitions.  *Complication severity not reported*  *Follow-up period is 30 days post operation.* | 30-day complication rate 27.0% | No Data  See Supplementary Table 3 for cost of readmission |
| **Nathan et al., 2015** | Multi-centre retrospective cohort study utilising Surveillance, Epidemiology,  and End Results cancer registry data linked with  Medicare claims | USA;  USD ($) 2011 | 31 191 patients undergoing colectomy for cancer;  2000 - 2007 | Open and laparoscopic colon resection techniques included and analysed as *one group* | Costs:  Hospital reimbursements defined as payments to index hospitalisation and readmissions initiated within 30 days post discharge.  Postoperative complications  Incidence of 30-day readmissions  *Postoperative follow-up period is 30-days post discharge* | 30-day Complication incidence = 32% | No Data  See Supplementary Table 3 for cost of readmission |
| **Keller et al., 2016** | Multi-centre retrospective cohort study utilising Truven Healthcare MarketScan commercial  claim data.  *(Conference abstract)* | USA;  USD ($),  assumed 2016 | 1 299 undergoing elective colectomies for colon cancer;  2013 | Open colon resection = 558 (43%)  Minimally invasive surgery = 741 (57%) | Costs:  Hospital reimbursements. Total and postoperative costs were calculated with readmissions capped at $100 000.  Postoperative complications  30-day Readmission rates.  *Follow-up period is 30 days post discharge.* | No Data | No Data  See Supplementary Table 3 for cost of readmission |
| **Widmar et al., 2016** | Single-centre retrospective cohort study  *(Conference abstract)* | USA;  assumed USD ($), assumed 2016 | 1 815 patients undergoing colectomy without ostomy;  2009 – 2014 | Colectomy without ostomy;  Surgical technique not specified | Costs:  Hospital reimbursements.  Hospital costs excluding day of surgery were estimated and converted to average Medicare reimbursements.  Postoperative complications  Patients categorised based on presence or absence of complications and by severity of complications as graded by the Clavien-Dindo classification system.  *Postoperative follow-up period was defined as 30 days after surgery* | Incidence of complications by Clavien-Dindo classification  No complication =1 028 (56.6%)  Complication grade 1 =231 (12.7%)  Complication grade 2 =390 (21.5%)  Complications grade 3+ =166 (8.8%) | Mean (SE) total 30-day reimbursement by complication severity  No complication = $3 520 (88) **[$3 756]**  Complication grade 1 = $5 570 (250) **[$5 943]**  Complication grade 2 = $ 7 610 (267) **[$8 119]**  Complication grade 3+ =17 124 (1120) **[$18 270]**  Mean (SE) total 90-day reimbursement by complication severity  No complication = $5 854 (160) **[$6 246]**  Complication grade 1 = $9 501 (470) **[$10 137]**  Complication grade 2 =$10 979 (379) **[$11 714]**  Complication grade 3+ =$27 174 (1766) **[$28 993]**  *31-90 days cost and post-discharge costs also reported.*  *Cost breakdown not available* |
| **Liu et al., 2017** | Multi-centre retrospective cohort study utilising the ACS-NSQIP and the Medicare Provider Analysis  and Review databases. | USA;  USD ($) 2012 | 19 089 patients undergoing elective colectomy;  2009-2012 | Colectomy;  Surgical technique not specified | Costs:  Hospital reimbursements.  Payments to hospitals by Centers for Medicare and Medicaid Services based on MS-DRG categories. Payments not directly related to care (geographic factors, education costs, and disproportionate  Share) were excluded.  Postoperative complications  Patients were categories based on presence or absence of postoperative complications and by complication type. Complications were identified using ACS NSQIP criteria.  *Complication severity not reported*  *Postoperative follow-up period is not specified* | Incidence of complications by complication type:  1 or more complication rate = 18.4%  UTI = 3.4%  SSI = 9.8%  DVT = 1.7%  Cardiac complications = 1.6%  Postoperative renal failure = 1.4%  Postoperative pneumonia = 2.5%  Unplanned intubation = 2.4%  Prolonged ventilation = 2.0% | Overall hospital payment increased by  $10 996 **[$12 263]** per complication  Mean reimbursement (95%CI) by complication type  Uncomplicated = $13 531.16 (13 390.63-13 673.15) **[$15 090]**  UTI = $15 867.66 (15 385.58-16 364.84) **[$17 696]**  Additional cost =$2 336.50 **[$2 606]**  SSI = $16 257.21 (15 940.01-16 580.72) **[$18 130]**  Additional cost =$2 726.05 **[$3 040]**  DVT = $19 260.51 (18 462.59-20 092.91) **[$21 479]**  Additional cost =5 729.35 **[$6 389]**  Cardiac complications = $18 984.67 (18 155.18-19 852.05) **[$21 172]**  Additional cost =5 453.51 **[$6 082]**  Postoperative renal failure = $20 054.42 (19 127.69-21 026.05) **[$22 365]**  Additional cost =6 523.27 **[$7 275]**  Postoperative pneumonia = $22 598.17 (21 763.12-23 465.25) **[$25 201]**  Additional cost =9 067.01 **[$10 112]**  Unplanned intubation = $18 290.67 (17 554.11-19 058.13) **[$20 398]**  Additional cost =4 759.51 **[$5 308]**  Prolonged ventilation = $27 626.21 (26 408.75-28 899.79) **[$30 809]**  Additional cost =14 095.05 **[$15 719]**  *Cost breakdown not available* |
| **Keller et al., 2017** | Multi-centre retrospective cohort study utilising Truven Healthcare MarketScan commercial  claim data. | USA;  USD ($)  assumed 2017 | 4 615 patients undergoing elective colon resection;  January 2013 – November 2013 | MIS colon resection =2 561 (55.5%)  Open colon resection =2 054 (44.5%) | Costs:  Hospital reimbursements for index admission and 30 days post discharge including all facility and professional payments. Readmission costs were capped at $100 000  Postoperative complications  30-day readmissions.  Patients categorised based on presence or absence of complications identified using ICD-9-CM codes.  *Postoperative follow-up period is 30-days post discharge* | Incidence of complications  Open colon resection = 52.8 %  MIS colon resection = 32.3 %  ; P-value <0.001 | No Data  See Supplementary Table 3 for cost of readmission |
| **Regenbogen et al., 2017** | Multi-centre cross-sectional cohort study using the Medicare provider analysis and review database | USA;  USD ($) 2012 | 639 943 patients out of which 189 229 patients were undergoing colon resection at 1 876 hospitals;  January 2009 – June 2012  Hospitals were categorised into shortest, medium and longest length of stay mode | Open and laparoscopic colon resection techniques included and analysed as *one group* | Costs  Hospital Reimbursements defined as payments to hospitals during 90-day surgical episode plus payments for readmissions within 90 days of discharge.  Postoperative complications  90-day readmission.  Patients categorised based on presence or absence of complications identified using the Complication Screening Project codes.  *Postoperative follow-up period is 90-days post discharge* | Incidence rate of any postoperative complication by index admission length of stay mode  Shortest (≤3 days) = 27.2%  Medium (4-6 days) = 31.0%  Longest (≥7 days) = 35.1% | No Data  See Supplementary Table 3 for cost of readmission |
| **Etter al., 2018** | Multi-centre retrospective cohort study utilising the Truven Health MarketScan Commercial and  Medicare Supplemental databases. | USA;  USD ($)  2015 | 64 532 patients undergoing colectomy;  2009 - 2013 | Open colectomy = 38 769 (60.1%)  Laparoscopic colectomy = 25 763 (39.9%)  Open and laparoscopic colon resection techniques included and analysed as *one group* for costing analysis | Cost:  Total hospital reimbursement. Definition not reported.  Postoperative complications  First rehospitalisation within one year of index hospitalisation due to adhesion related complications (small bowel obstruction, adhesiolysis or ileus).  *Postoperative follow-up period is one-year post discharge* | Overall incidence of readmissions = 15 933 (24.7%)  Adhesion related complication readmissions =3 704 (5.7%); 23.2% of all first readmissions | No Data  See Supplementary Table 3 for cost of readmission |
| **Negative cost coverage** | | | | | | | |
| **Langelotz et al., 2017** | Single-centre retrospective cohort study utilising the Institute for the Hospital Remuneration System database  *(Conference abstract)* | Germany,  Euro (€) assumed 2017 | 460 patients undergoing colonic resections;  2010-2015 | Colonic resection;  surgical technique not specified | Costs:  Cost coverage effect of complications.  Cost definition not given.  Postoperative complications  Patients categorised based on the presence or absence of SSI and anastomotic insufficiency.  *Complication severity not reported*  *Postoperative follow-up period not specified.* | Incidence of SSI =56 (12.2%)  Incidence of anastomotic insufficiency =25 (5.4%)  Combined incidence of SSI and anastomotic insufficiency = 75 (16.3%) | Median negative cost coverage effect of complications  Without SSI or anastomotic insufficiency = –668€ **[$786]**  With SSI only = –6 823€ **[$8 026]**  With anastomotic insufficiency only = –2 659€ **[$3 128]**  *Cost breakdown not available* |
| *Costs in bold,* ***[$$$]****, have been converted and inflated to February 2019 $USD*  *Weighted mean cost was calculated by the authors of the review from reported complication costs*  ACS-NSQIP: American College of Surgeons-National Surgical Quality Improvement Project; SSI: Surgical Site Infection; POI: Postoperative Ileus; PPC: Postoperative Pulmonary Complications; DVT: Deep Vein Thrombosis; UTI: Urinary Tract Infection; CVA: Cerebral Vascular Accident; ORAE: Opioid Related Adverse Event; ICD-9-CM: International Classification of Diseases, Ninth Revision, Clinical Modification; MIS: Minimally Invasive Surgery; SD: Standard Deviation; SE: Standard Error; 95% CI: 95% Confidence Interval; IQR: Interquartile range; USD: United States Dollar; NZD: New Zealand Dollar; MS-DRG: Medicare Severity - Diagnosis Related Group; DRG: Diagnosis Related Group; ERAS: Enhanced Recovery After Surgery; HALS: Hand-Assisted Laparoscopic Surgery | | | | | | | |
