# Supplementary material for: Financial burden of postoperative complications following colonic resection: A systematic review
Source: Medicine (Baltimore). 2021 Jul 9;100(27):e26546. doi: 10.1097/MD.0000000000026546 (PMC8270623; doi:10.1097/MD.0000000000026546)
Supplement: Supplemental Digital Content [file medi-100-e26546-s003.docx]

**Supplementary Table 3** - **Summary and results of secondary outcomes of included studies reporting on resource use/costs of postoperative complications following colonic resection surgery.**

| **Author, year** | **Length of stay and associated costs** | **Readmission and associated costs** | **Mortality and associated costs** | **Indication for surgery and associated costs** | **Surgical urgency and associated costs** | **Surgical technique and associated costs** |
| --- | --- | --- | --- | --- | --- | --- |
| **Studies reporting on hospital costs** | | | | | | |
| **Braga et al., 2010** | No Data | No Data | No Data | No Data | No Data  *All patients were elective admissions* | Complication incidence  Laparoscopic colon resection =16 (11.9%)  Open colon resection = 27 (20.1%)  ; P-value = 0.094  Mean additional cost of complications  Laparoscopic colonic resection = €1 478.63 **[$2 290]**  Open colonic resection = €2 662.89 **[$4 123]**  *Cost of complication calculated by dividing the total cost of complications in the laparoscopic and open groups by the number of patients who experienced a complication.* |
| **Sammour et al., 2010** | No Data | Readmission incidence  ERAS group = 12%  Control group =14%  ; P-value=0.766  Mean cost of readmission = $4 688.0 **[$3 946]** | Mortality incidence  ERAS group = 0 (0%)  Control group = 2 (4%)  Impact of complications on mortality not reported | No Data | No Data  *All patients were elective admissions* | No Data |
| **Bloom et al., 2011** | Mean LOS in laparoscopic colectomy  With PPC =10.9 days  Without PPC =9.5 days; P-value<0.0001  Mean LOS in open colectomy  With PPC = 13.1  Without PPC =10.9  ; P-value <0.0001  Cost of length of stay not reported. | No Data | No Data | No Data | No Data | PPC incidence  Laparoscopic colectomy =9.4%  Open colectomy = 26.3%  Mean total cost of PPC for laparoscopic colectomy  With PPC = $28 759 **[$33 010]**  Without PPC= $24 013 **[$27 563]**  ; P-value<0.0001  Mean total cost of PPC for open colectomy  With PPC = $34 053 **[$39 087]**  Without PPC = $25 524 **[$29 297]**  ; P-value <0.0001 |
| **Delissovoy et al., 2011** | Additional mean (95%CI) LOS with SSI = 9.72 (9.70-9.74) days  Cost of length of stay not reported. | No Data | No Data | No Data | No Data | No Data |
| **Ramamoorthy 2012** | Mean LOS  With ORAE= 7.7 days  Without ORAE = 6.2  ; P-value<0.0001  Cost of length of stay not reported. | No Data | No Data | No Data | No Data | No Data  *All patients underwent laparoscopic colectomy* |
| **Kashimura et al, 2012** | Mean (SD) LOS  With SSI = 30.6 (36.2) days  Without SSI = 12.6 (5.9) days.  Difference = 18.0 [95%CI - 10.8 to 25.2] days  ; P-value<0.001  Cost of length of stay not reported. | There were no readmissions to any of the participating institutions. | One patient died in hospital | No Data | No Data | Incidence of complications  No Data  Patients with and without SSI were case-matched:  Laparoscopic group = 25/50 (50%)  Open group = 77/154 (50%)  Mean cost (SD) in open colectomy  With SSI = $10 047 (10 651) **[$11 204]**  Without SSI $4 482 (3 105) **[$4 998]**  Difference = $5 565 [95%CI: 3 053 – 8 077] **[$6 206]**  ; P-value<0.001  Mean cost (SD) in laparoscopic colectomy  With SSI = $10 475 (20 116) **[$11 682]**  Without SSI =$3 656 (2 329) **[$4 077]**  Difference = $6 819 [95%CI: -1 529 to 15 166] **[$7 605]**  ; P-value=0.049 |
| **Kalogera et al., 2013** | No Data | No Data | No Data | No Data  *All patients had large bowel resection for primary ovarian cancer* | No Data | No Data |
| **Thacker et al., 2014** | Mean (± SD) LOS  With POI = 10.0 (±8.5)  Without POI = 5.5 (±4.7)  ; P-value <0.0001  Cost of length of stay not reported. | 30-day readmission incidence  With POI = 13.7%  Without POI = 9.4%  ; P-value <0.0001  Cost of readmission not reported. | Mortality incidence  With POI = 1.6%  Without POI = 0.6%  ; P-value <0.0001  Cost of mortality not reported | No Data | No Data  All patients were elective admissions | No Data |
| **Asgeirsson et al., 2014** | No Data | Readmission incidence laparoscopic resection = 65 (9.9%)  Open resection = 131 (17.1%).  Mean (±SD) cost of readmission  Laparoscopic resection = $14 358 (± $9 972) **[$15 516]**    Open resection = $24 220 (±$24 835) **[$26 173]** | No Data | No Data | Urgent/emergent  admissions were associated with increased numbers of complications  per patient (P-value =0.010) as compared to elective/preadmit admissions. Actual incidence of complications per group not reported.  Mean (±SD) cost for laparoscopic resection  Elective/preadmit patients = $8 014 (± 4850) **[$8 660]**  Urgent/emergent admissions = $13 997 (±8 847) **[$15 126]**  Mean (±SD) cost for open resection Elective/preadmit patients = $12 041 (±10 915) **[$13 012]**  Urgent/emergent admissions = $19 319 (±19 434) **[$20 877]** | Open colectomy was associated with an increased number of complications per patient as compared to laparoscopic colectomy (1.15 ± 0.08 vs 0.26 ± 0.03; P-value <0.001).  Mean (± SD) cost with no complications  Laparoscopic resection= $7 738 (± 4 150) **[$8 362]**  Open resection = $10 934 (± 12 498) **[$11 816]**  ; P-value<0.001  Mean (± SD) cost in laparoscopic group:  No complication = $7 739 (± 4 150) **[$8 363]**  1 complication = $11 878 (± 7 266) **[$12 836]**  2 complications = $12 465 (± 4 153) **[$13 470]**  3+ complications = $24 549 (± 11 816) **[$26 528]**  *Weighted mean additional cost*  *Any complication = $7 407.60* ***[$8 005]***  Mean (± SD) cost in open group:  No complication = $10 935 (± 12 498) **[$11 817]**  1 complication = $13 989 (± 9 570) **[$15 117]**  2 complications = $18 300 (± 11 218) **[$19 776]**  3+ complications = $30 529 (± 21 704) **[$32 991]**  *Weighted mean additional cost*  *Any complication = $10 663.74* ***[$11 524]*** |
| **Flynn et al., 2014** | Entire cohort mean (±SD) LOS  Without complication = 7.3 (±2.4) days  With complication = 15.6 (±7.8) days  Difference =8.3 days  ; P-value <0.001  Matched sample (n=100; 50 uncomplicated and 50 complicated) mean (± SD) LOS  Without complication = 7.4 (±2.1) days  With complication =15.9 (±7.5) days  Difference =8.5 days  ; P-value <0.001  Cost of length of stay not reported | No Data  (Readmissions within 30 days were combined with the total LOS and cost of index admission) | 1/276 deaths occurred  Cost of mortality not reported | No Data | No Data | No Data  *All patients had open partial colectomy with anastomosis* |
| **Manecke et al., 2014** | Mean (± SD) LOS  With complication =23.1 (±25.2) days  Without complication = 9.6 (± 8.2) days  ; P-value<0.001  Cost of length of stay not reported. | No Data | Mortality rate (%)  With complication = 15.2 %  Without complications = 2.6%  ; P-value<0.001  Cost of mortality not reported | No Data | No Data | No Data |
| **Gan et al., 2015** | Median LOS in open colectomy  With POI = 12 days  Without POI = 8 days  ; P-value <0.0001  Median LOS in laparoscopic colectomy  With POI = 8 days  Without POI = 4 days  ; P-value <0.0001  Cost of length of stay not reported. | 30-day readmission rate in open colectomy  With POI = 17.7%  Without POI = 15.4%  ; P-value<0.0001  30-day readmission rate in Laparoscopic colectomy  With POI = 13.4%  Without POI = 8.9%  ; P-value <0.0001  Cost of readmission not reported. | No Data | No Data | No Data | Incidence of postoperative ileus  Open colectomy = 8 303/40 250 (20.6%)  Laparoscopic colectomy = 2 577/17 698 (14.6%)  Median cost for Laparoscopic colon resection:  With POI = $17 505 **[$18 933]**  Without POI = $12 521 **[$13 543]**  ; P-value <0.0001  Median cost for open colon resection:  With POI = $24 078 **[$26 043]**  Without POI = $17 044 **[$18 435]**  ; P-value <0.0001 |
| **Knechtle et al., 2015** | Average LOS by complication count  0 complication =9.5 days  1 complication =14.0 days  2 complications =20.2 days  3 complications =23.5 days  4+ complications =27.4 days  Trend with increasing number of complications performed using overall F-test and test for β1 is statistically significant (P-value <0.0001)  Cost of length of stay not reported. | 30-day readmission incidence  Total 30-day readmissions = 45 (12%)  0 complication = 20 (7.6%)  1 complication = 15 (20.3%)  2 complications = 8 (32.0%)  3 complications = 2 (18.2%)  4+ complications = 0 (0.0%)  No P-value  Cost of readmission not reported. | No Data | No Data | No Data | No Data |
| **Phothong et al., 2015** | Mean (± SD) LOS  With SSI = 23.5 (±9.8) days  Without SSI =9.8 (±3.8) days  ; P-value <0.001  *LOS data only available for SSI in open surgery group.*  Cost of length of stay not reported. | No Data | No Data | No Data  All patients had sigmoidectomy for sigmoid cancer. | No Data | Complication incidence by complication type:  Anastomosis leakage - HALS 0 (0%) as compared to open surgery 2 (4%); P=0.155  Bowel ileus - HALS 3 (6%) as compared to open 2 (4%); P=0.560  SSI - HALS 0 (0%) as compared to Open 6 (12%); p=0.012  Lung complication - HALS 0 (0%) as compared to Open 2 (4%); p=0.560  Cost data available for SSI in open surgery group only (see Supplementary Table 2). |
| **Manzanares et al., 2016** | Total mean (±SD) hospital stay  With complication = 17.3 (±9.3) days  Without complication = 8.9 (±5.6) days  ; P-value <0.0001  Cost of length of stay not reported. | No Data | No Data | No Data  *All patients had a diagnosis of colon cancer* | No Data | No Data |
| **Healy et al., 2016** | No Data | No Data | No Data | No Data | No Data | No Data |
| **Zogg et al., 2016** | Risk-adjusted predicted mean (95%CI) additional LOS by complication type  Any Complication = 3.76 (3.66–3.87) days  Mechanical Wound = 4.72 (4.36–5.08) days  Infection = 5.55 (5.35–5.76) days  Urinary = 1.41 (1.15–1.66) days  Pulmonary = 4.36 (4.12–4.60) days  Gastrointestinal= 3.79 (3.65–3.92) days  Cardiovascular = 2.84 (2.60–3.08) days  Systemic = 2.96 (2.50–3.42) days  Surgical = 2.07 (1.80–2.34) days  Cost of length of stay not reported. | No Data | Risk-adjusted predicted odds ratio (95%CI) for in-hospital mortality by complication type  Any Complication = 4.98 (4.29–5.77)  Mechanical  Wound = 3.81 (2.58–5.61)  Infection = 3.97 (3.16–4.98)  Urinary = 1.86 (1.21–2.85)  Pulmonary = 8.00 (6.65–9.62)  Gastrointestinal= 2.22 (1.88–2.62)  Cardiovascular = 5.63 (4.62–6.86)  Systemic = 7.03 (4.86–10.15)  Surgical = 2.75 (2.00–3.78)  Cost of mortality not reported | Weighted percentage of any complication:  Colon cancer = 18.1%  Diverticulosis, diverticulitis = 14.9%  Benign neoplasm of the colon = 14.7%  Regional enteritis, ulcerative colitis =17.7%*; P-value <0.01*  *See Supplementary Table 2 for cost by principal diagnosis data* | No Data  All patients were elective admissions | Weighted percentage incidence of any complication by surgical technique:  Laparoscopic = 12.67%  Open or other = 20.39%  ; P-value<0.01  Breakdown by complication type reported  *See Supplementary Table 2 for cost data* |
| **Martin et al., 2017** | No Data | No Data | 30-day mortality incidence  Overall =88/7 102 (1.2%)  Open colectomy = 69/4 685 (1.5%)  Laparoscopic colectomy = 19/2 417 (0.8%)  ; P-value = 0.70  Impact of complications on mortality not reported | No Data  *All patients had a diagnosis of benign colon polyps* | No Data  All patients were elective admissions | Complication incidence  Open colectomy =1 026/4 685 (21.9%)  Laparoscopic colectomy =372/2 417 (15.4%)  ; P-value =0.65  Median (IQR) cost for open colectomy  Cost of colectomy = $22 712 ($16 621-32 508) **[$23 641]**  With complication = $32 460 ($20 842-54 665) **[$33 788]**  Median (IQR) cost for laparoscopic colectomy  Cost of colectomy = $20 697 ($14 905-29 268) **[$21 544]**  With complication = $27 639 ($19 752-45 748) **[$28 770]** |
| **Zogg et al., 2018** | No Data  *LOS impact of complications for colon surgery specifically is not reported* | No Data | No Data  *Mortality for colon surgery specifically is not reported* | No Data  *All patients had a diagnosis of colon cancer* | No Data | No Data |
| **Studies reporting on hospital charges** | | | | | | |
| **Fukuda et al., 2012** | Mean (95%CI) postoperative LOS  With SSI =19.6 (19.3–19.9) days  Without SSI =12.2 (12.0–12.4) days  Difference =7.4 (7.2–7.5) days  ; P-value<0.001  Mean (95%CI) additional LOS in open colon surgery  Superficial SSI =4.5 (4.4–4.5) days  ; P-value<0.001  Deep SSI = 6.8 (6.7–6.9) days  ; P-value<0.001  Space/organ SSI =7.8 (7.6–7.9) days  ; P-value<0.001  Mean (95%CI) additional LOS in laparoscopic colon surgery  Superficial SSI =4.8 (4.6–4.9) days  ; P-value<0.001  Deep SSI =10.3 (10.0–10.5) days  ; P-value = 0.002  No patient developed space/organ infection in laparoscopic colon surgery.  Cost of length of stay not reported | No Data | No Data | Principal diagnosis  Tumour =71/1 308  Cancer =964/1 308  Invagination/Ileus =73/1 308  Other =200/1 308  Adjusted odds ratio (95%CI) of SSI  Tumour =1  Cancer = 1.9 (0.7–5.1)  Invagination/Ileus = 2.1 (0.6–7.5)  Other = 3.0 (1.1–8.3)  No principal diagnosis was statistically associated with increased risk of SSI.  Cost by principal diagnosis not given. | Non-Emergent n=1 115/1 308  Emergent n=193/1 308  Adjusted odds ratio (95%CI) of SSI  Non-Emergent = 1  Emergent = 1.0 (0.5–1.9)  No surgical urgency status was statistically associated with increased risk of SSI.  Cost by surgical urgency status not given | A significantly lower SSI rate  for colon surgery was found in the laparoscopic group when compared with the open surgery group (6.9% vs. 17.3%; P<0.001; χ^2^ test)  Mean (95% CI) increase in postoperative charge for open surgery  With superficial SSI = $853 (834–873) **[$995]**  ; P-value = 0.002  With deep SSI = $1 847 (1 808–1 886) **[$2 155]**  ; P-value = 0.024  With space/organ SSI = $2 003 (1 957–2 046) **[$2 337]**  ; P-value<0.001  Mean (95% CI) increase in postoperative charge for laparoscopic surgery  With superficial SSI = $739 (717–763) **[$862]**  ; P-value<0.001  With deep SSI = $1 672 (1 619– 1 726) **[$1 950]**  ; P-value = 0.016  No patient developed space/organ infection in laparoscopic colon surgery. |
| **Vaid et al., 2012** | Median LOS in open colon resection  Without complication = 6 days  With complication = 10 days  Median LOS in laparoscopic colon resection  Without complication = 5 days  With complication = 9 days  Cost of length of stay not reported. | No Data | Mortality rate  Laparoscopic colon resection =87 (1.70%)  Open colon resection =1 438 (2.40%)  ; P-value=0.001  Impact of complications on mortality not reported  Cost of mortality not reported | No Data  *All patients had a diagnosis of colon cancer* | No Data  *All patients were elective admissions* | Incidence of complications  Open colon resection = 27.1%  Laparoscopic colon resection = 18.9%  ; P-value<0.001  *Breakdown by complication type provided*  Median charge in laparoscopic colon resection:  Without complication =$39 030 **[$43 526]**  With complication =$58 382 **[$65 108]**  ; P-value < 0.001  Median charge in open colon resection  Without complication =$39 152 **[$43 662]**  With complication =$62 221 **[$69 389]**  ; P-value < 0.001 |
| **Studies reporting on hospital reimbursements** | | | | | | |
| **Birkmeyer et al., 2010** | No Data | Mean payments for readmissions  Average reimbursement when present = $12 023 **[$14 025]**  Average reimbursement overall = $1 740 **[$2 030]**  Incidence of readmission not reported | No Data | No Data  *All patients had a diagnosis of colon cancer* | No Data | No Data |
| **Wick et al., 2011** | Mean (95% CI) LOS  With SSI = 9.5 days (9.0-10.0)  Without SSI = 8.1 (8.0-8.2) days  ; P-value <0.001  Cost of length of stay not reported. | Readmission incidence  With SSI= 27.8%  Without SSI = 6.8%  ; P-value<0.001  Cost of readmission not reported. | No Data | Principal diagnosis  Colon cancer =3 909 (55.7%)  Diverticulitis =2 817 (40.1%)  Inflammatory bowel disease =294 (4.2%)  Impact of indication for surgery on odds ratio (95%CI) of developing SSI with colon cancer as a reference  Colon cancer 1 [Reference]  Diverticulitis =0.98 (0.83-1.15)  Inflammatory bowel disease =1.27 (0.88-1.84)  No surgical indication was associated with increased risk of SSI  Cost by principal diagnosis not reported | No Data | Open colectomy as compared with laparoscopic colectomy was associated with an increased risk of SSI (odds ratio=1.57; 95% confidence interval: 1.25-1.97)  Incidence and cost of SSI by surgical technique not reported |
| **Birkmeyer et al., 2012** | No Data | Mean payments for readmissions  Low risk hospitals = $1 322 **[$1 474]**  High risk hospitals = $1 736 **[$1 936]**  Incidence of readmission not reported | Mortality rates ranged from 2.11% to 10.92%  Impact of complications on mortality not reported | No Data  *All patients had a diagnosis of colon cancer* | No Data | No Data |
| **Regenbogen et al., 2012** | No Data | Mean payments for readmissions  Low-volume hospitals = $1 564 **[$1 744]**  High-volume hospitals= $1 391 **[$1 551]**  Incidence of readmission not reported | 30-day mortality incidence  Low-volume hospitals = 6.4%  High-volume hospitals= 6.0%  *Impact of complications on mortality not reported* | No Data  *All patients had a diagnosis of colon cancer* | No Data | No Data |
| **Lawson et al., 2013** | No Data | Mean (95%CI) 30-day readmission rate  Overall readmission rate = 13.4%  With complication =28.4%  Without complication =8.1%%  Mean reimbursement for a 30-day readmission  With complication = $13 419 **[$14 730]**  Without complication = $7 954 **[$8 731]** | No Data | No Data | No Data | No Data |
| **Nathan et al., 2015** | No Data | 30-day Readmission  Readmission incidence =12%  Payment for readmissions = $1 937 **[$2 223]** – 6% of total episode payment | 30-day Mortality  Mortality incidence = 3.0%  Impact of complications on mortality not reported | No Data  *All patients had a diagnosis of colon cancer* | No Data | No Data |
| **Keller et al., 2016** | No Data | Readmission rate  Open colectomy = 10.93%  Laparoscopic colectomy = 6.61%  ; P-value=0.0165  Mean reimbursement for readmissions  Open colectomy = $3 151**[$3 362]**  Laparoscopic colectomy = $1 676 **[$1 788]**  ; P-value=0.0309 | No Data | No Data  *All patients had a diagnosis of colon cancer* | No Data  *All patients were elective admissions* | No Data  See readmissions |
| **Widmar et al., 2016** | No Data | No Data | No Data | No Data | No Data | No Data |
| **Liu et al., 2017** | No Data | No Data | No Data | No Data | No Data  *All patients were elective admissions* | No Data |
| **Keller et al., 2017** | No Data | 30-day readmission incidence (%)  Open resection =11.54%  MIS resection = 8.28%;  ; P-value= 0.0013  Mean readmission-associated reimbursements  Open colon resection = $3 055 **[$3 180]**  MIS resection = $2 514 **[$2 617]**  ; P-value = 0.1858 | No Data | No Data | No Data  *All patients were elective admissions* | Incidence of complications  Open colon resection = 52.8 %  MIS colon resection = 32.3 %  ; P-value <0.001  See readmissions |
| **Regenbogen et al., 2017** | No Data | Payments for readmissions  Hospitals with shortest index admission LOS = $2 606 **[$2 906]**  Hospitals with longest index admission LOS = $2 887 **[$3 220]**  Incidence of readmission not reported | No Data | No Data | No Data | No Data |
| **Etter al., 2018** | No Data | Overall incidence of readmissions = 15 933 (24.7%)  Adhesion related complication (ARC) readmissions =3 704 (5.7%); 23.2% of all first readmissions  Mean (SD) total reimbursement  ARC readmission = $29 802 (43 037) **[$32 234]**  Non-ARC readmission = $22 476 (36 120) **[$24 310]**  ; P-value<0.0001 | No Data | Incidence of ARC related readmission based on principal diagnosis  Cancer =919/20 134 (4.6%)  Diverticulitis =1 026/17 875 (5.7%)  IBD =348/2 130 (16.3%)  Diverticulosis =93/1 742 (5.3%)  OR (95%CI) of ARC-related readmission for IBD with cancer as a reference = 1.69 (1.43–1.99)  ; P-value<0.0001  Cost by principal diagnosis not given. | No Data | Incidence of readmission  Open  Total readmission n=11 627/38 769 (30%)  ARC related readmission n= 2 897/38 769 (7.5%)  Laparoscopic  Total readmission n= 4 306/ 25 763 (16.7%)  ARC related readmission n=807/ 25 763 (3.1%)  Laparoscopic resection had 68% lower odds of an ARC-related readmission as compared to open surgery (OR=0.42, 95%CI:0.39–0.46)  ; P-value<0.0001  No cost data available |
| **Negative cost coverage** | | | | | | |
| **Langelotz et al., 2017** | Median LOS  With SSI = 30 days  Without SSI = 11 days  With anastomotic insufficiency but without SSI = 18 days  Cost of length of stay not reported. | No Data | No Data | No Data | No Data | No Data |
| *Costs in bold,* ***[$$$]****, have been converted and inflated to February 2019 $USD*  *Weighted mean cost was calculated by the authors of the review from reported complication costs*  LOS: Length of Stay; SD: Standard Deviation; 95%CI: 95% Confidence Interval; IQR: Interquartile range; SSI: Surgical Site Infection; POI: Post-Operative Ileus; HALS: Hand-Assisted Laparoscopic Surgery; MIS: Minimally Invasive Surgery; ARC: Adhesion-Related Complication; IBD: Inflammatory Bowel Disease; ERAS: Enhanced Recovery After Surgery; PPC: Postoperative Pulmonary Complication; ORAE: Opioid Related Adverse Event | | | | | | |
